# Transcriptional dynamics during *Rhodococcus erythropolis* infection with phage WC1

**DOI:** 10.1186/s12866-024-03241-4

**Published:** 2024-04-01

**Authors:** Dana L. Willner, Sudip Paudel, Andrew D. Halleran, Grace E. Solini, Veronica Gray, Margaret S. Saha

**Affiliations:** 1 Data Science Program, William & Mary, Williamsburg, VA USA; 2 Department of Biology, William & Mary, Williamsburg, VA USA; 3 Wyss Institute, Harvard University, Cambridge, MA USA; 4 Atalaya Capital Management, New York, NY USA; 5 California Institute of Technology, Pasadena, CA USA; 6 Georgetown University School of Medicine, Washington, DC USA

**Keywords:** *Rhodococcus*, Phage, WC1, Transcriptome, RNA-Seq

## Abstract

**Background:**

Belonging to the Actinobacteria phylum, members of the *Rhodococcus* genus thrive in soil, water, and even intracellularly. While most species are non-pathogenic, several cause respiratory disease in animals and, more rarely, in humans. Over 100 phages that infect *Rhodococcus* species have been isolated but despite their importance for *Rhodococcus* ecology and biotechnology applications, little is known regarding the molecular genetic interactions between phage and host during infection. To address this need, we report RNA-Seq analysis of a novel *Rhodococcus erythopolis* phage, WC1, analyzing both the phage and host transcriptome at various stages throughout the infection process.

**Results:**

By five minutes post-infection WC1 showed upregulation of a CAS-4 family exonuclease, putative immunity repressor, an anti-restriction protein, while the host showed strong upregulation of DNA replication, SOS repair, and ribosomal protein genes. By 30 min post-infection, WC1 DNA synthesis genes were strongly upregulated while the host showed increased expression of transcriptional and translational machinery and downregulation of genes involved in carbon, energy, and lipid metabolism pathways. By 60 min WC1 strongly upregulated structural genes while the host showed a dramatic disruption of metal ion homeostasis. There was significant expression of both host and phage non-coding genes at all time points. While host gene expression declined over the course of infection, our results indicate that phage may exert more selective control, preserving the host’s regulatory mechanisms to create an environment conducive for virion production.

**Conclusions:**

The *Rhodococcus* genus is well recognized for its ability to synthesize valuable compounds, particularly steroids, as well as its capacity to degrade a wide range of harmful environmental pollutants. A detailed understanding of these phage-host interactions and gene expression is not only essential for understanding the ecology of this important genus, but will also facilitate development of phage-mediated strategies for bioremediation as well as biocontrol in industrial processes and biomedical applications. Given the current lack of detailed global gene expression studies on any *Rhodococcus* species, our study addresses a pressing need to identify tools and genes, such as *F6* and *rpf*, that can enhance the capacity of *Rhodococcus* species for bioremediation, biosynthesis and pathogen control.

**Supplementary Information:**

The online version contains supplementary material available at 10.1186/s12866-024-03241-4.

## Background

*Rhodococcus* species comprise a genus of gram-positive actinomycetes in the Actinobacteria phylum best known for their ability to metabolize environmental pollutants and the capability to produce desirable biological compounds such as steroids [1–5]. Due to a high tolerance to toxic substrates, the capacity to degrade a wide range of organic and xenobiotic substances, and the ability to produce biosurfactant, *Rhodococcus* spp. are ideal candidates for the bioremediation of contaminated sites [3]. Members of the genus have a broad geographic range, and have been isolated from a variety of environments, most often soil including at contaminated sites, but also from freshwater, wastewater, sediment, air, and crude oil [2, 6–8]. They have also been found in host-associated systems, and while most species are benign, some can cause infections in plants [9] and animals [10], and more rarely in humans [11, 12].

Bacteriophage, viruses which infect bacteria, are the most abundant biological entities on earth, and are generally considered to be ten times as numerous as their hosts in any environment [13]. Dynamics in natural ecosystems are mediated by phage-host interactions, and phage predation has the potential to be exploited for use in industrial and biomedical applications. *R. opacus* phage Toil has been used as a bioextraction agent for biodiesel production, forcing the release of triacylglycerols from its host upon lytic infection [14]. Phage of *Rhodococcus* and other related Actinobacteria isolated from wastewater have been suggested for use as biocontrol agents to reduce foaming in activated sludge [15, 16]. Phage YF1, which can infect *R. equi*, *R. erythropolis*, *R. rhodochrous*, and *R. opacus*, was used to identify potential new antimicrobial targets in its hosts, serving as a model for novel target discovery in other related organisms [17].

A significant number of Rhodococcal phage have been isolated and sequenced; NCBI and the Actinobacteria Phage Database currently contain entries for 74 sequenced phage of 4 *Rhodococcus* species [18]. The most numerous of these are phage that infect *Rhodococcus erythropolis* RIA-643, many of which have been characterized in conjunction with the Science Education Alliance Phage Hunters Advancing Genomics and Evolutionary Science (SEA-PHAGES) program [19, 20]. The majority of these sequenced phage have been classified in cluster CA, a group of temperate phage which share a common genomic architecture [20, 21]. Cluster CA phage have regulatory structures and a large subset of protein coding genes similar to cluster A mycobacteriophages [20]. Phage gene expression during infection and, in some cases, corresponding host responses have been described at the molecular level for mycobacteriophage [22–24]. However, despite their importance, exploration of transcriptional dynamics remains largely unexplored for *Rhodococcus* phage and their hosts.

Here, we present Winter Compost 1 (WC1), a new phage of *Rhodococcus erythropolis RIA-*643 isolated in Williamsburg, Virginia in 2018. We characterize WC1 at the genomic level, and analyze temporal transcriptional profiles of both phage and host during infection using RNA-seq. The genome of WC1 was typical of cluster CA phage with high (> 90%) levels of nucleotide similarity and between coding sequences. During host infection WC1 genes were expressed following a temporal program of early, middle, and late, which was also mirrored by the spatial organization of transcribed regions in the genome. WC1 infection results in a dramatic suppression of host metabolism and a notable disruption of metal ion, particularly, iron homeostasis. By 120 min, 80% of the transcripts map to phage genes. Only 13% of host genes are differentially expressed. This work provides insight into how a cluster CA phage alters the global transcription program of its host during infection, and may apply more broadly to other Rhodococcal phage-host systems. A more detailed understanding of these phage-host interactions can facilitate development of phage-mediated strategies for bioremediation as well as biocontrol in industrial processes and biomedical applications.

## Methods

### *Rhodococcus erythropolis* host strain and WC1 bacteriophage isolation

*Rhodococcus erythropolis* RIA-643 [BUCSAV 57.1] was obtained from ATCC (ATCC® 15,903™). Cells were initially grown in Middlebrook 7H9 media with AD supplement (10%), carbenicillin (50 µg/ml), cyclohexamide (10 µg/ml), and calcium chloride (1 mM). For RNA-Seq experiments carbenicillin and cyclohexamide were omitted. Winter Compost 1 Phage (WC1) was isolated from a compost sample at 37.282925 N, 76.664688 W in 2014 using a standard enrichment protocol [18, 23]. Briefly the soil sample was incubated with a culture of *Rhodococcus erythropolis* in media for 24 h at 37 C. The sample was filtered with a 0.2 μm PES filter and 100 µl of the filtrate and 500 µl of a *R. erythropolis* culture was combined with 7H9 top agar and plated on LB plates. Plaques were visible within 24 h; plaques were subjected to three rounds of purification to ensure a plaque pure sample.

### WC1 bacteriophage DNA isolation and sequencing

Following plaque purification, high titer lysate was obtained by flooding host plates displaying web lysis with 1X phage buffer (10mM Tris, pH 7.5, 10 mM MgSO4, and 0.4% w/v NaCl). To isolate phage DNA, the lysate was treated with nuclease mix (0.8 U/ml DNase I and 100 µg/ml RNase) with 12.5mM MgCl_2_ at 37 °C for 30 min in order to remove bacterial DNA. Phage capsid was digested using proteinase K (500 µg/mL) and a detergent (0.5% SDS) after stopping DNase activity using a bivalent cation chelator (2mM EDTA) at 55 °C for 60 min. Phage genomic DNA was extracted using Phenol:Chloroform:Isoamyl alcohol (25:24:1), and precipitated using sodium acetate (0.3 M) and ice cold ethanol. Finally, the DNA was collected by centrifuging 13,000 rpm for 10 min; the DNA pellet was washed with 70% ethanol and re-suspended in nuclease free water. Phage genomic DNA was sequenced using the Ion Torrent PGM Sequencer system with standard library preparation according to the manufacturer’s protocol for 150 bp reads (Ion PGM Sequencing Kit, 314 Chip v2).

### WC1 genome assembly and annotation

Raw WC1 reads were quality checked using FastQC v0.11.5 [25]. Reads were trimmed using Trimmomatic v3.6 [26] with default parameters resulting in 1.2 million reads, giving 7500X coverage of the genome. *De novo* genome assembly was performed using the CLC Microbial Genomics Module to obtain major contigs with a minimum of 100X coverage, and finishing was performed with Consed [27] and the CLC Microbial Genomics Module (Qiagen) according to the protocols provided at [28]. Initial gene prediction (features) were obtained using DNA Master [29], which makes use of Glimmer [30], GeneMarkS [31], and Aragorn (Laslett and Canback 2004). In the process of gene-by-gene refinement, the features were then manually modified, deleted, or inserted following the Guiding Principles of Bacteriophage Genome Annotation (06/16/2018) available via SEA PHAGES [19]. Decisions were made based on coding potential (GeneMark Version 2.5p), RBS score (using SD Scoring Matrix Kibler6 and Spacing Weight Matrix Karlin Medium available in DNA Master), length of ORF, number of gaps or overlapping nucleotides, and BLASTp match (evalue < 10^− 4^). The tRNA genes predicted by DNA Master were further confirmed using ARAGORN v1.2.38 [32] and tRNAscan-SE v. 2.0 [33]. For protein assignments, each gene was evaluated using HHPred [34] and BLASTp [35]. Context of the functions of the flanking genes (synteny) was examined using Phamerator [36].

### Phage comparative genomics

Genomes for 38 previously isolated phage of *R. erythopolis* RIA-643 were obtained from Genbank (accession numbers and cluster IDs appear in Table S1). This includes 34 phages from Cluster CA (temperate), 3 from cluster CB (lytic), and 1 from cluster CE (unknown lifestyle) of *Rhodococcus* phage as delineated by phagesdb [18]. GC content and pairwise nucleotide similarity between WC1 and all genomes was determined using the online tool JSpecies WS [37] with the ANIM MUMmer-based option. Genome architectures were obtained from Phamerator [36] for comparison with WC1.

### One-step growth curve experiment

*R. erythropolis* RIA-643 log phase liquid-culture and WC1 bacteriophage were combined at a multiplicity of infection of 1:10. Cells were incubated at 37 °C for 5 min to allow phage adsorption, and then centrifuged for 3 min at 5,000G at 4 °C. Pelleted cells were washed to remove unadsorbed phage particles using 1 mL room temperature phage buffer (10mMTris pH 7.5, 10mMMgSO4, 4% w/v NaCl). Cells were subjected to three rounds of washing, each of which was followed by centrifugation at 5,000G for 3 min at 4 °C. Final bacterial pellets were then resuspended in 40 ml Middlebrook 7H9 media. For the duration of the experiment, cells were incubated in with shaking 37 °C. 200 µL samples were taken at 5,

30, 36, 50, and 60 min and then every subsequent 30 min over a 4 h period and were serially diluted in phage buffer. Samples were then added to 500 µL of uninfected *R. erythropolis*, incubated for 5 min at room temperature, and plated for measurement of viral titer to assess the length of the latent period.

### RNA preparation and sequencing

To obtain RNA samples, 15 mL of *R. erythropolis* cell were collected prior to addition of phage. For the 5, 30, 60, and 120 min time points, 15 mL of phage-infected *R. erythropolis* cells were. Cells were centrifuged at 4000 G at 4 °C for 10 min. The resulting pellets were flash frozen in liquid nitrogen and transferred to -80 °C for storage. One ml of TRIzol (Life Technologies) was used to resuspend frozen pelleted cells, and total RNA was extracted as previously described [23]. The RiboZero kit for gram-positive bacteria (Illumina) was used to deplete ribosomal RNA from RNA extraction samples following manufacturer’s instructions. Following depletion, RNA library size and effectiveness of rRNA depletion was checked using a Bioanalyzer 2100 (Agilent). rRNA-depleted RNA was then prepared for sequencing using the TruSeq Stranded RNA-Seq Kit (Illumina) and sequenced on an Illumina Miseq (150 bp reads) according to the manufacturer’s instructions generating a total of 55,302,168 paired-end reads. All reads used for this study had a Q35 score. Raw RNA-Seq reads can be accessed via NCBI’s Short Read Archive at project accession number SRP154435.

### Differential expression analysis

Sequence length, quality, and composition for raw reverse-stranded RNA-Seq reads which contained both bacteria and bacteriophage transcripts evaluated using FastQC v0.20.0 [25]. Trimming and filtering was performed using Trimmomatic v3.9 [26] with the parameters SLIDINGWINDOW:4:15 TRAILING:20 MINLEN:75 AVGQUAL:20 based on review of the FastQC outputs. Libraries were aligned to phage and host genomes using HISAT2 v2.2.1 [38] excluding multi-mapping reads. Read coverage was assessed using the bamtools coverage tool [39], with subsequent read counts normalized by library size and averaged over replicates. The resulting alignments were mapped to features for the phage and host genomes using FeatureCounts [40] from the SubRead Anaconda package version 2.0.3, with option -s 2 to only assign reads if they align reverse-stranded to a feature, -p to count paired end reads as a single fragment, and the options -O and --fraction to count overlapping reads and assign fractional amounts of reads according to the number of features that a read overlaps.

Differential expression (DE) analysis was conducted using the R package DESeq2 (version 1.38.3) [41], using *ashr* for LFC shrinkage [42]. Pairwise comparisons between all time points were conducted using individual Wald tests. Results were filtered to adjusted *p*-value of 0.05 for Wald tests, as well as minimum absolute log_2_ fold change of approximately 0.58, representing a minimum absolute fold change of 1.5, which is in accordance with guidelines established by Schurch et al. [43].Hierarchical clustering of phage genes was performed using scikit-learn AgglomerativeClustering with complete linkage. The optimal number of clusters was determined by creating a dendrogram containing the full tree.

### Host RNA-seq functional enrichment analysis

GO terms were assigned to *R. erythopolis* RIA-643 genes using a combination of bioinformatic strategies which allowed for cross-referencing and confirmation of term assignments. Automated analyses were carried out using eggNOG-mapper (http://eggnog-mapper.embl.de/) [44], and the command line version of InterProScan v5.63-95 (https://www.ebi.ac.uk/interpro/about/interproscan/) [45, 46]. GO terms were also assigned using a modification of the approach available at https://github.com/enormandeau/go_enrichment. Specifically, all bacterial coding sequences were translated using gffread [47] and compared to the full Swissprot [48] database (available from: ftp://ftp.ncbi.nlm.nih.gov/blast/db/swissprot) using BLASTp (e-value < 0.001, max of 1 target sequence) [35]. BLAST hits were filtered using the parameters outlined in Rost (1999) which use require an adjusted cutoff percent similarity based on the length of residues aligned. Annotations for significant hits were retrieved from UniProtKB [48]via the RESTful API and were parsed for GO terms. Terms assigned to genes by more than one method were cross-validated, and the union of all assignments was used as the final set of GO terms, resulting in 4846 annotated coding sequences. All annotated bacterial coding sequences were functionally annotated with KEGG terms using the KEGG Orthology Based Annotation System Intelligent version (KOBAS-i) . The annotation program was run using the closest KEGG annotated reference organism, *Rhodococcus sp.008*, and resulted in 6233 annotated and 602 unannotated coding sequences. Enrichment analysis for GO and KEGG terms was performed in R with ClusterProfiler (version 4.6.2) on log_2_ fold-change values for all genes that were not filtered by DESeq2 independent filtering. The enricher function was used for GO, and the enrichKEGG function for KEGG. Non-coding RNAs found to be DE in the host were characterized using the Rfam database [49]. GO terms were condensed based on term similarity using the command line tool GO-Fig. [50].

## Results

### WC1 genome architecture is concordant with other temperate *R. erythropolis* phage

The WC1 genome contained 46,439 base pairs, with a GC content of 58.6%. The length and GC content were consistent with cluster CA phage of *R. erythropolis* RIA-643, which are known to be temperate (Figure S1) [20]. WC1 had high average nucleotide identity to other *R. erythropolis* cluster CA phages, ranging from a low of 92.7% (Partridge) to a high of 98.6% (Erik). Within the WC1 genome, 66 protein coding genes were identified, of which, 35 (53%) were annotated with putative functions (Figure S2). Three tRNAs were annotated on the far left arm of the genome, approximately 1.5 kb from the origin, as seen previously in cluster CA phage [20].

Overall, the genomic content and architecture of WC1 shows markedly high synteny with previously described temperate phages of *R. erythropolis* RIA-643. As described in [20], this includes, from left to right, the three tRNAs previously mentioned, a cluster of genes related to virion structure and assembly, a putative immunity repressor, and a genetic cluster with functions necessary for replication and regulation (Figure S3). There are 16 copies (13 forward, 3 reverse complement) of the 13mer 5’-YGWCTATTGTCAA-3’ primarily (75%) in intergenic regions of WC1 (Figure S2). These regions have previously been described in *R. erythropolis* cluster CA phage as well as cluster A mycobacteriophage, and have a putative regulatory function, maintaining lysogeny by binding the immunity repressor [20, 51].

### Phage transcripts dominate during the infection time course

**Fig. 1:**
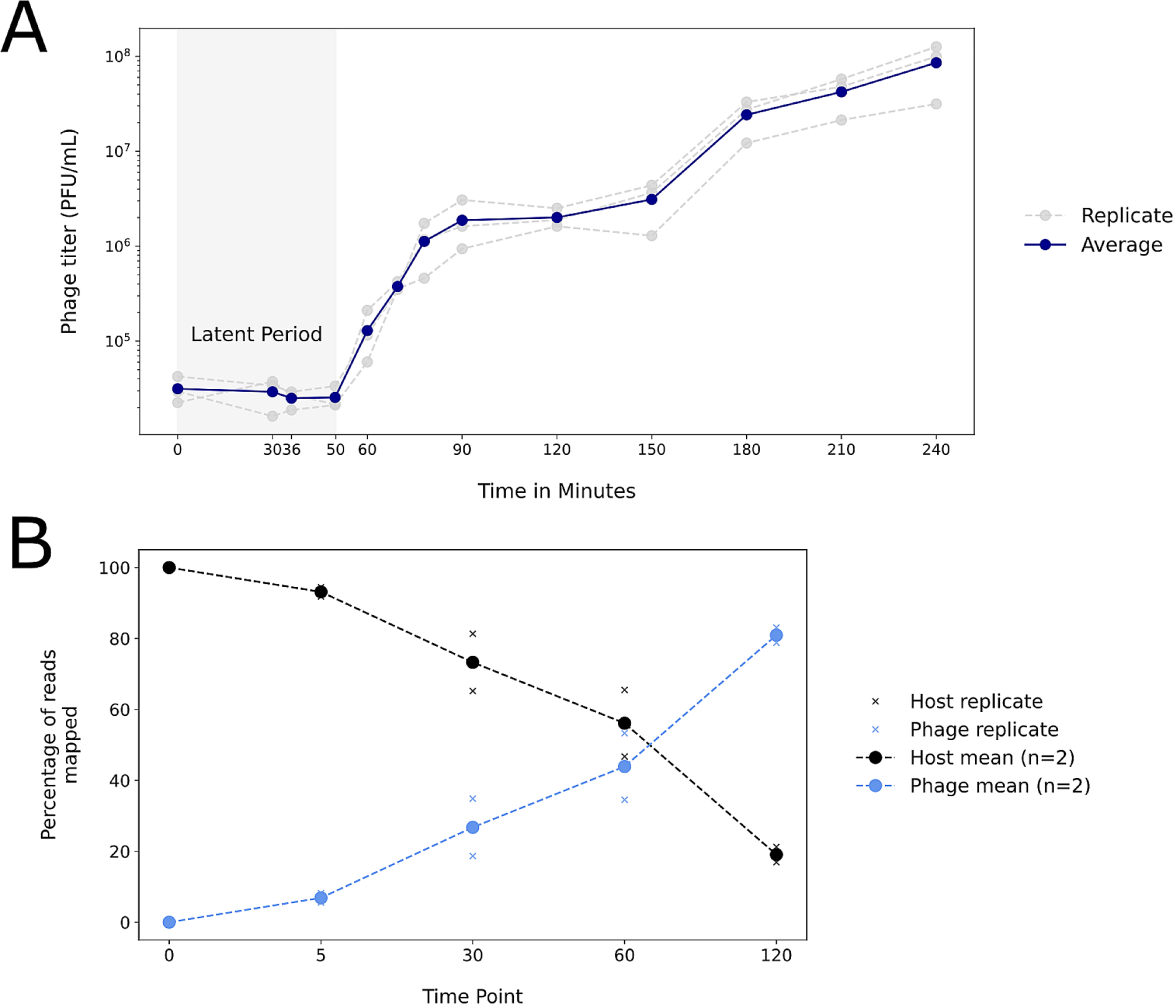
**A**: Phage titer during host infection. Grey lines and circles represent individual replicate experiments. The blue line shows the average. **B**: Percentage of reads at each time point mapped to host versus phage genomes. Solid circles show averages of two replicates and the x labels show individual replicates – in most cases these are close to the mean and thus do not appear

Dual RNA-sequencing of the host and the infecting phage was performed prior to infection (0 min) and at four timepoints up to 120 min after infection. Time points for RNA-seq were chosen based on exploration of the phage lifecycle via a one-step growth experiment (Fig. 1A). Specifically, the 5 and 30 min time points represent early and late phases in the latent period respectively; the 60 min time point corresponds to the lysis phase, and the 120 min time point lies at the point of what appears to be a second burst, indicative of another round of infection (Fig. 1A).

Overall, for all replicates at all time points, the proportion of total RNA-Seq reads mapped to either the phage or host genome ranged from 94.72 to 97.92% (Table S2). Over the time course of the infection, the proportion of total RNA-Seq reads aligning to the host showed a marked decrease while reads aligning to the phage genome showed the opposite trend, increasing to approximately 81% of total aligned reads at 120 min (Fig. 1B). A small number of reads aligned to phage features at time 0, but these represented less than 0.02% of total reads aligned (Table S3).

### Transcriptional activation of phage genes follows temporal and spatial patterns

**Fig. 2:**
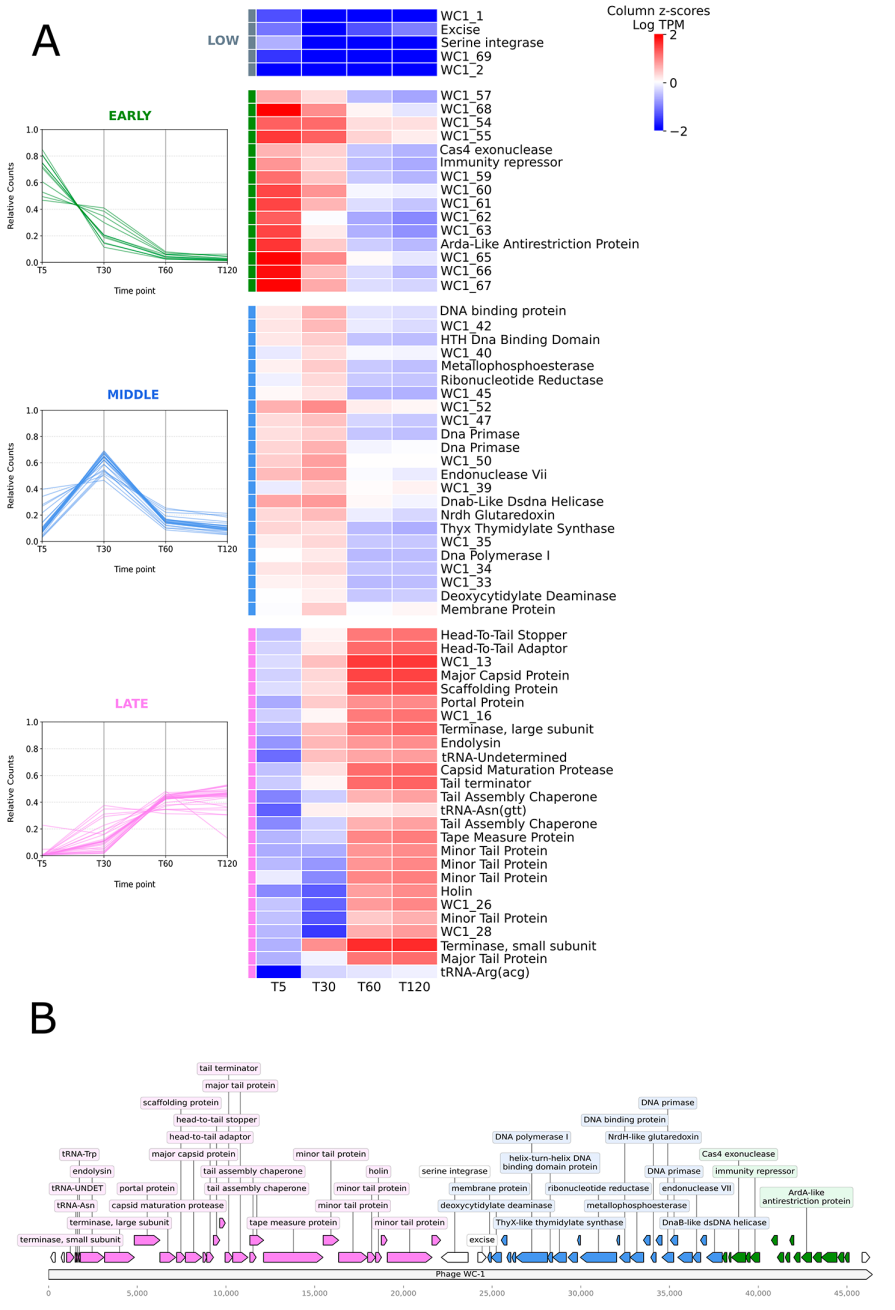
Temporal and spatial patterns of transcriptional activation in WC1. (**A**) Phage expression patterns over time. Parallel coordinate plots (left) show relative counts of phage transcripts for Early, Middle, and Late genes. The counts are transcripts per million (TPM) normalized internally for each gene, i.e. with respect to the lowest expression level for that individual gene. The heatmap (right) shows log TPM averaged at each time point and subsequently standardized. The standardization is performed by column. (**B**) Genomic locations of early, middle, and late genes in WC1. Colors used in the genome map correspond to those in A

Phage genes demonstrated temporal expression patterns which largely corresponded to specific regions of the phage genome (Fig. 2). The 69 phage genes were grouped using hierarchical clustering of normalized log counts (see Methods), which were indicative of three distinct temporal patterns: early, middle, and late stage genes. The stage of each gene corresponds in general to its peak expression. Genes in the early, middle, and late classes tended to be spatially clustered near each other in the genome (Fig. 2B). A fourth cluster contained 5 genes with relatively lower expression across all timepoints: 3 corresponding to proteins of unknown function, and the phage serine integrase, and excise genes (Fig. 2A).

Five minutes post-infection, the most highly expressed genes were localized to the right arm of the phage genome (Fig. 2). Early genes demonstrated high expression levels five minutes after infection, which were sustained or minimally decreased at 30 min, and then subsequently declined (Fig. 2). WC1_68 (a hypothetical protein with unknown function) was the most highly expressed transcript at the five minute time point. The majority of these genes corresponded to proteins of unknown function. The cluster also included a Cas exonuclease, an immunity repressor, and an anti-restriction protein.

At 30 min post-infection, the majority of phage genes exhibited high levels of expression. Middle genes specifically reached peak expression at the 30 min time point and represented genes to the left of the early cluster (Fig. 2). This group contained several genes related to DNA synthesis and replication: deoxycytidylate deaminase, ThyX thymidylate synthase, ribonucleotide reductase phosphoesterase, NrdH glutaredoxin, and a dsDNA helicase. Genes involved in phage genome replication were also highly expressed, including DNA polymerase I, two DNA primases, a DNA-binding protein, and a `phage membrane protein. The other 10 genes in the middle cluster encoded proteins of unknown function.

By 60 min post-infection, the left arm of the phage genome demonstrated markedly higher levels of expression (Fig. 2). The late stage cluster demonstrated peak expression at 60 min, which was sustained for nearly all genes at 120 min, while the genes in the other clusters showed decreased expression at both 60 and 120 min. The late genes were comprised largely of phage structural proteins, phage assembly and packaging proteins, and host lysis and degradation proteins including an endolysin and a holin. The remaining five protein-encoding genes in this cluster included the terminase, both the small and large subunits, and several which had unknown functions; the terminase, small subunit was the most highly expressed genome feature at both 60 and 120 min post-infection. Three phage-encoded tRNAs also clustered with the late group due to sustained high expression at 60 and 120, but displayed similarly high expression at 30 min.

**Fig. 3:**
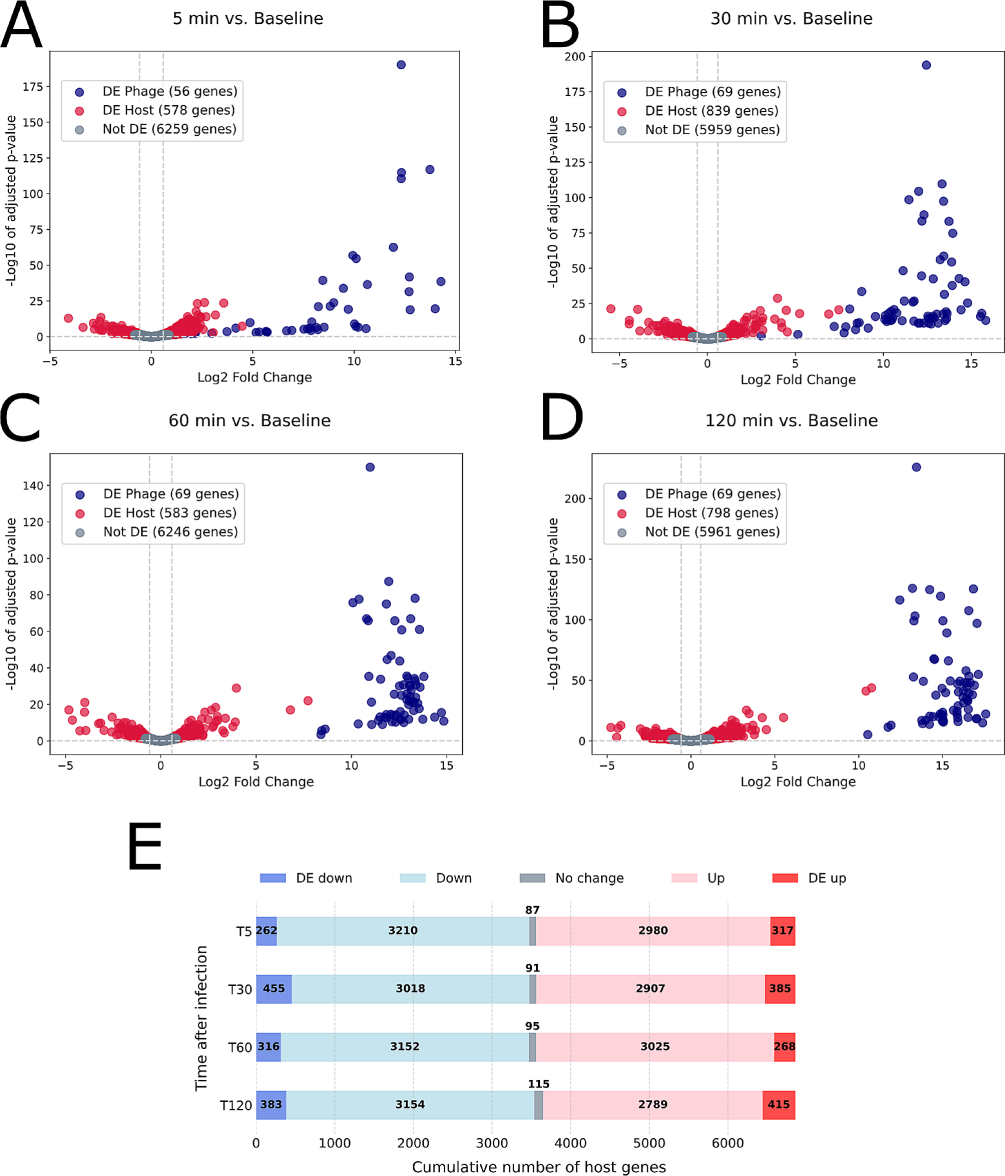
Differentially expressed (DE) genes over the time course of infection. **A-D**: Volcano plots for each time point compared to baseline. Each point on the volcano plot is based on 2 replicates. Vertical lines indicate Log_2_ Fold Change of 0.58 and − 0.58, which corresponds to a fold change of 1.5. The horizontal line indicates p of 0.05. **E**: Counts of up and down-regulated host genes as compared to the 0 min baseline. Genes considered DE had FC greater than 1.5 and *p*-values less than 0.05

Differential expression (DE) analysis further reinforced these patterns as well as the dominance of phage transcripts during infection (Fig. 3). By 5 min post-infection, 56 phage genes showed significant (Wald test, *p*-value < 0.05, FC > 1.5) increases in expression when compared to the 0 min baseline (Fig. 3A). The 13 genes that were not significant belonged to the left arm of the WC-1 genome, specifically WC-1 genes 2–7, 13, 19,20, 22, and 25–26, with the single exception of WC1_69. By 30 min post-infection, all 69 phage genes were up-regulated as compared to baseline, and this was sustained at 60 and 120 min (Fig. 3B-D). By 120 min post-infection all phage genes had log_2_ fold changes greater than 1.5 and *p*-values less than 0.00001. Sequential comparisons over the time course of infection mirror sequential activation of phage genomic regions (Figure S4; Figure S5).

### Non-coding regions of WC-1 were the most highly transcribed genomic loci post-infection

**Fig. 4:**
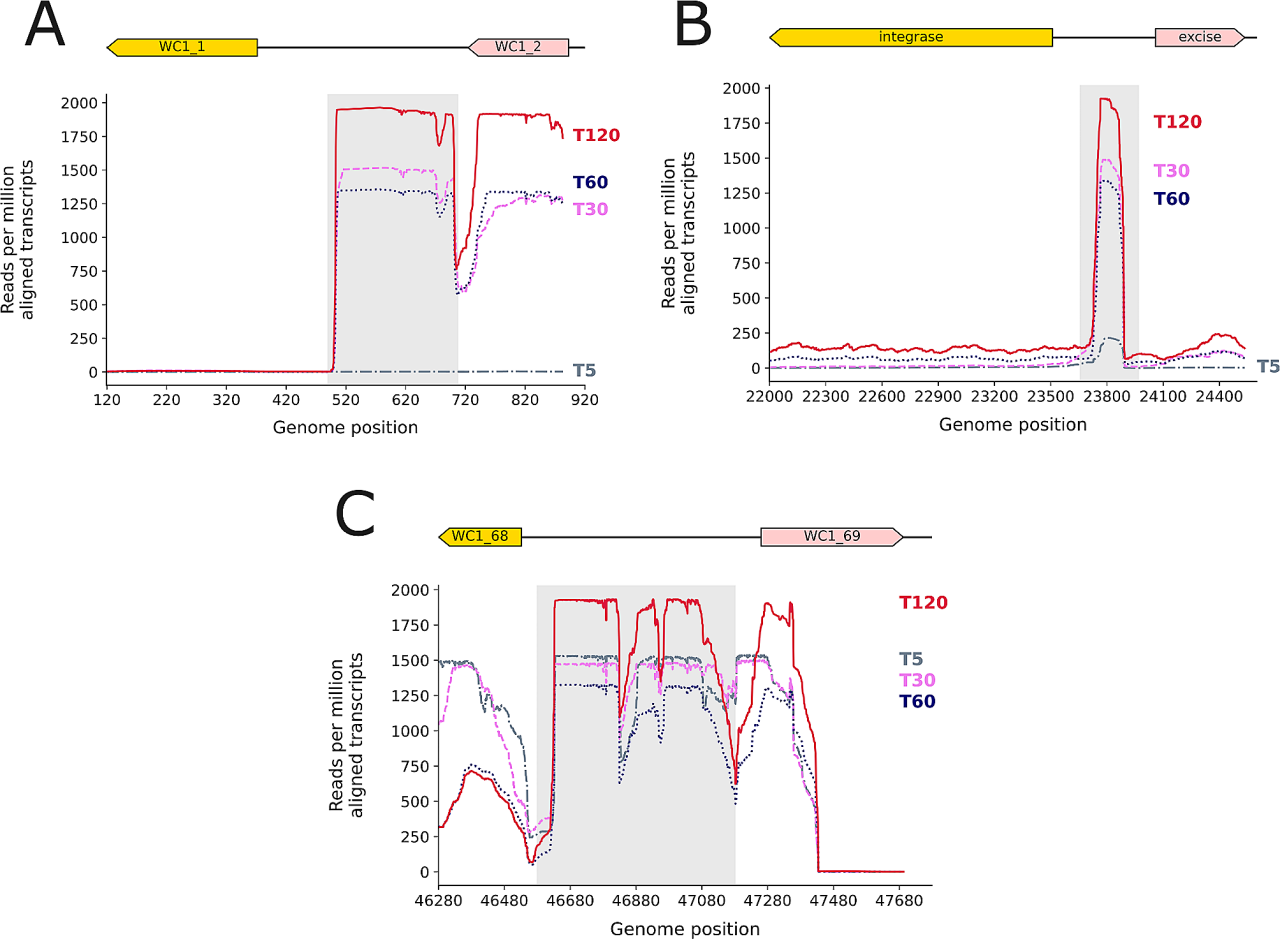
Transcribed intergenic non-coding regions in WC1. The y axis shows reads normalized per million aligned transcripts in each individual library and then averaged for each set of replicates at each genomic location

Phage WC-1 contains three transcribed intergenic non-coding regions: between WC1_1 and WC1_2 (nc_1), between WC1_29 and WC1_30 (nc_2), and between WC1_68 and WC1_69 (nc_3). All three regions were most highly expressed 120 min after infection relative to other time points (Fig. 4). At 120 min as well as at 30 and 60 min post-infection, these regions had the highest relative read coverage when compared to the rest of the genome (Figure S6). Of these, region nc_1 had the highest normalized mapped read counts at 30, 60, and 120 min relative to other time points (Fig. 4A), but also relative to all other genome features (Figure S6). This region was not active at 5 min post-infection, which is in concordance with gene expression on the left arm of the genome, which was most active at later in the time course of infection. Region nc_2 (Fig. 4B; Figure S6) showed minimal transcription at 5 min, corresponding to low activation in the central region of the genome overall. In contrast, region nc_3 showed high levels of transcription at 5 min, as did all features in the right arm of the genome (Fig. 4C; Figure S6).

### A core set of host genes were differentially expressed throughout infection

Of the 6928 identified genes of RIA-643, a total of 874 unique host transcripts were differentially expressed over the time course of the infection when each time point was compared to the baseline (Fig. 3). Overall, the proportion of up- versus down-regulated host genes, whether DE or not, were nearly equal, with slightly more suppression than activation at all time points (Fig. 3E). The number of host DE genes varied between time points (Fig. 3), with slightly more up-regulated DE genes at 5 and 120 min, and slightly more down-regulated at 30 and 60 min (Fig. 3E). A core set of 201 genes and 2 non-coding RNAs were DE at every time point with fold change greater than 1.5 as compared to time 0 (Figures S7, S8, S9; Table S4). Of these, 37 encoded proteins of unknown functions.

Two non-coding RNAs were up-regulated at all timepoints versus the baseline (Figure S8). During the course of phage infection in RIA-643, the small RNA F6 (DVG80_03131) reached peak expression at 30 min with lower expression levels at 60 and 120 min. The F6 small RNA (sRNA) in RIA-643 shares 100% identity with 8 other identified F6 sRNAs in the Rfam database, which are found in other strains of *R. erthryopolis* and other *Rhodococcus* species. This sRNA is part of Rfam family F6 (RF01791). F6 has been shown to have a regulatory function in *Mycobacterium* species, associated with and up-regulated during stress responses [52–54]. The ydaO/yuaA leader (DVG80_05852) sequence was most highly expressed at 120 min versus the baseline. This RNA was identical to the ydaO/yuaA leader sequence in *R. erythropolis* PR4, and is part of the ydaO-yuaA (RF00379) Rfam family. ydaO-yuaA is a riboswitch that responds to cyclic di-AMP, serving a regulatory role in transcription [55, 56].

There were 85 universally up-regulated protein-encoding genes (Table S4). These included 5 transcription factors and 13 ribosomal proteins. The most highly up-regulated gene at all time points, with the largest positive log_2_ Fold Change of all genes (not just the shared set), encoded a protein containing a DUF3542 domain (DVG80_03545). An adjacent gene also of unknown function (DVG80_ 03546) was the second most universally up-regulated (Figure S7). DUF3542 is an immunoglobulin-like domain that is related to extracellular immunoprotective functions [57], and InterProScan results were consistent with this, indicating that the DUF3542 domain of DVG80_03545 is flanked by a putative signal peptide, and is predicted to lie outside of the cytoplasmic space. Four of six resuscitation-promoting factor (rpf) genes were up-regulated and DE throughout the course of infection (RIA-643 genes 01821, 01260, 05851, 06126). These genes encode peptidoglycan glycosidases and have redundant and overlapping functionality.

Among the 116 universally down-regulated genes, there were 16 transcriptional regulators (Table S4). Five genes involved in both the TCA and glycoxylate cycles, as well as a sixth gene, malate synthase, which is unique to the glycoxylate cycle, were significantly suppressed at all time points during infection, while three additional related genes were DE at only a subset of time points (Table S5). Other TCA cycle genes which were not DE were generally down-regulated (i.e. negative log_2_FC as compared to baseline) at all time points (Table S5). Three moeY genes involved in molybdopterin biosynthesis (DVG80_01963, 06751, 06752) were part of the core set, while a fourth related gene, a FAD-binding molybdopterin dehydrogenase (DVG80_02060) was significant at 5, 30, and 60 min, and borderline significant (log_2_FC: -0.94, *p*-value: 0.06) at 120 min. A universal stress response gene (DVG80_06017) also showed decreased expression at all time points.

### WC1 infection modulated expression of genes involved in metal ion homeostasis

Five genes related to membrane transport of metal ions were universally up-regulated as part of the host core set (Table S4; Table S6). This included two of three contiguous genes encoding the substrate-binding protein (DVG80_04450) and permease (DVG80_04452) of a metal ABC transporter. The putative third component, the ATPase (DVG80_04451), was significantly up-regulated at all time points except 5 min, where it was borderline significant (log_2_FC: 0.87, *p*-value: 0.05). Based on homology to to ZnuABC of *Rhodococcus erythropolis VKPM Ac-1659* [6], and MntABC of *Rhodococcus erythropolis PR4* this transporter was predicted to mediate the influx of zinc and/or manganese. A divalent metal cation transporter (DVG80_05365), and an iron transporter homologous to EfeU, and thus predicted to mediate iron influx [58], were also up-regulated. A third related over-expressed gene, DVG80_03824, was homologous to fhuD of *Rhodococcus erythropolis VKPM Ac-1659*, which encodes the siderophore binding domain of an ABC transporter [6]. Other genes encoding components of siderophore ABC transporters were DE at all timepoints except 5 min; three of these were up-regulated while a fourth was down-regulated (Table S6).

In contrast to core up-regulated genes largely related to iron and metal import, genes involved in metal export and sequestration were strongly down-regulated throughout the time course of infection (Figure S9; Table S6). A gene (DVG80_04092) encoding a UPF0016 domain containing protein had the largest negative log_2_ fold change at all time points except 120 min, where it was the second largest. UPF0016 domain containing proteins have been shown to be involved in manganese export [59]. The expression of a VIT family protein (DVG80_05717) decreased over the time course of infection, from a log_2_FC of -2.55 to -4.18 by 120 min (Figure S9; Tables S4 and S6). VIT family proteins are ferritins that export iron and/or manganese, and have been characterized in numerous organisms, including various *Rhodococcus* species [60], other bacterial lineages [61], fungi [62], eukaryotic parasites [63, 64], and plants [65]. A bacterioferritin (DVG80_04076) was significantly under-expressed at all time points, reaching strongest suppression at 60 min post infection (Figure S4; Table S6). A second bacterioferritin in the RIA-643 genome was not DE, but rather exhibited constitutively low expression levels including at baseline. Similarly, genes homologous to components of an iron-regulated transcriptional repressors, furA (DVG80_03017), furB (DVG80_00409), and ideR (DVG80_01378) were not among down-regulated DE genes. The fur homologs exhibited low transcriptional levels at all time points, while expression of the ideR homolog slightly suppressed at all time points. Cytochrome d ubiquinol oxidase subunit I (DVG_05658) showed a markedly strong drop in expression from 5 min (log_2_FC: -1.21) to 30 min (log2FC: -4.45), and this decrease continued over time. The adjacent gene encoding subunit II (DVG_05659) was significantly and strongly down-regulated at all time points except 5 min. The cytochrome d oxidase contains iron conjugated structurally in heme [66].

### Functional enrichment of host genes reflected the phage WC1 lifecycle

**Fig. 5:**
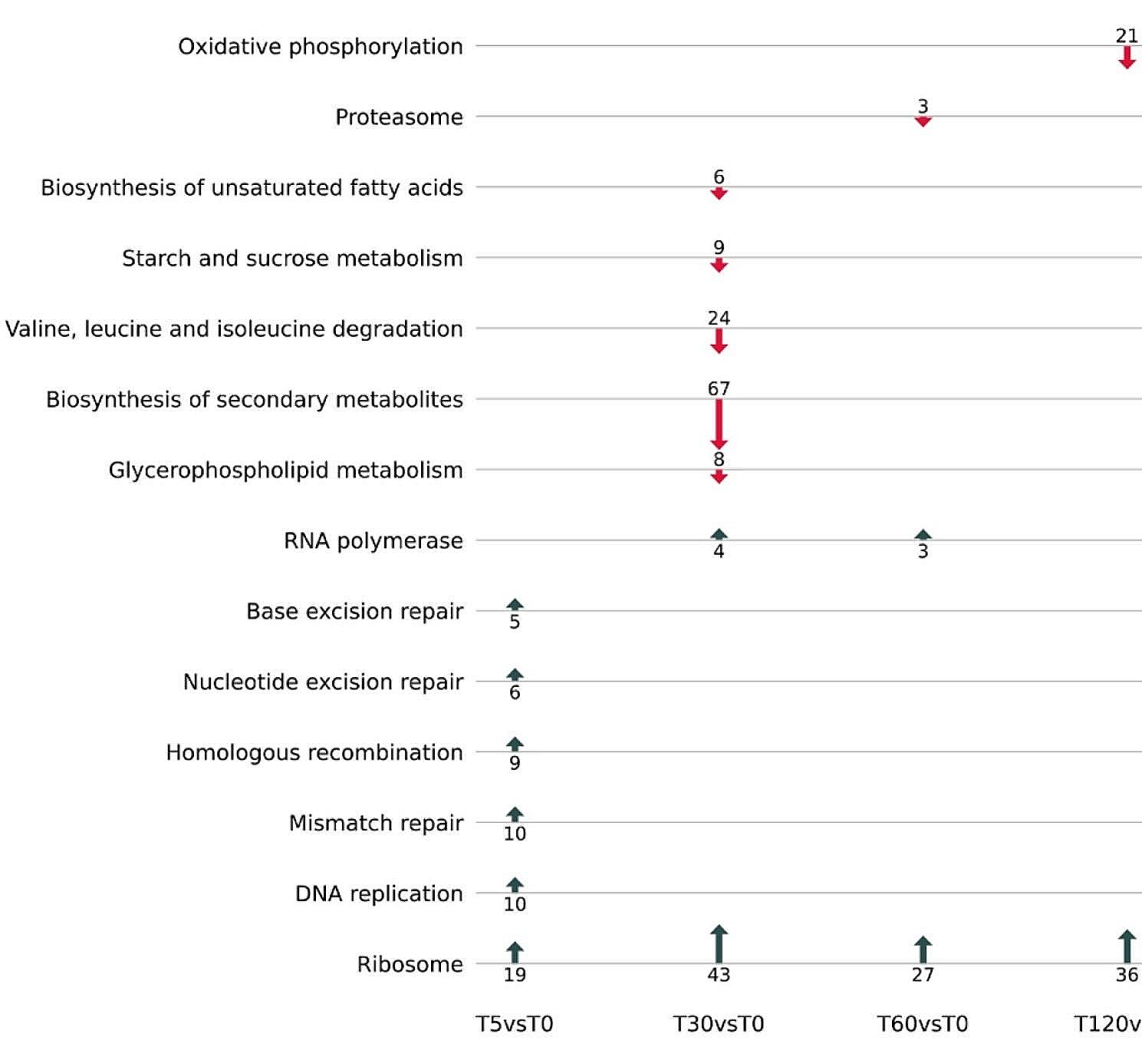
Enriched KEGG pathways during time course of infection as compared to baseline. The direction of the arrows indicate if the DE genes were up-regulated or down-regulated, and the numeric values indicate the number of genes. Only DE genes with absolute log_2_ fold change greater than or less than − 0.58 were included in the analysis to reflect a fold-change of 1.5

**Fig. 6:**
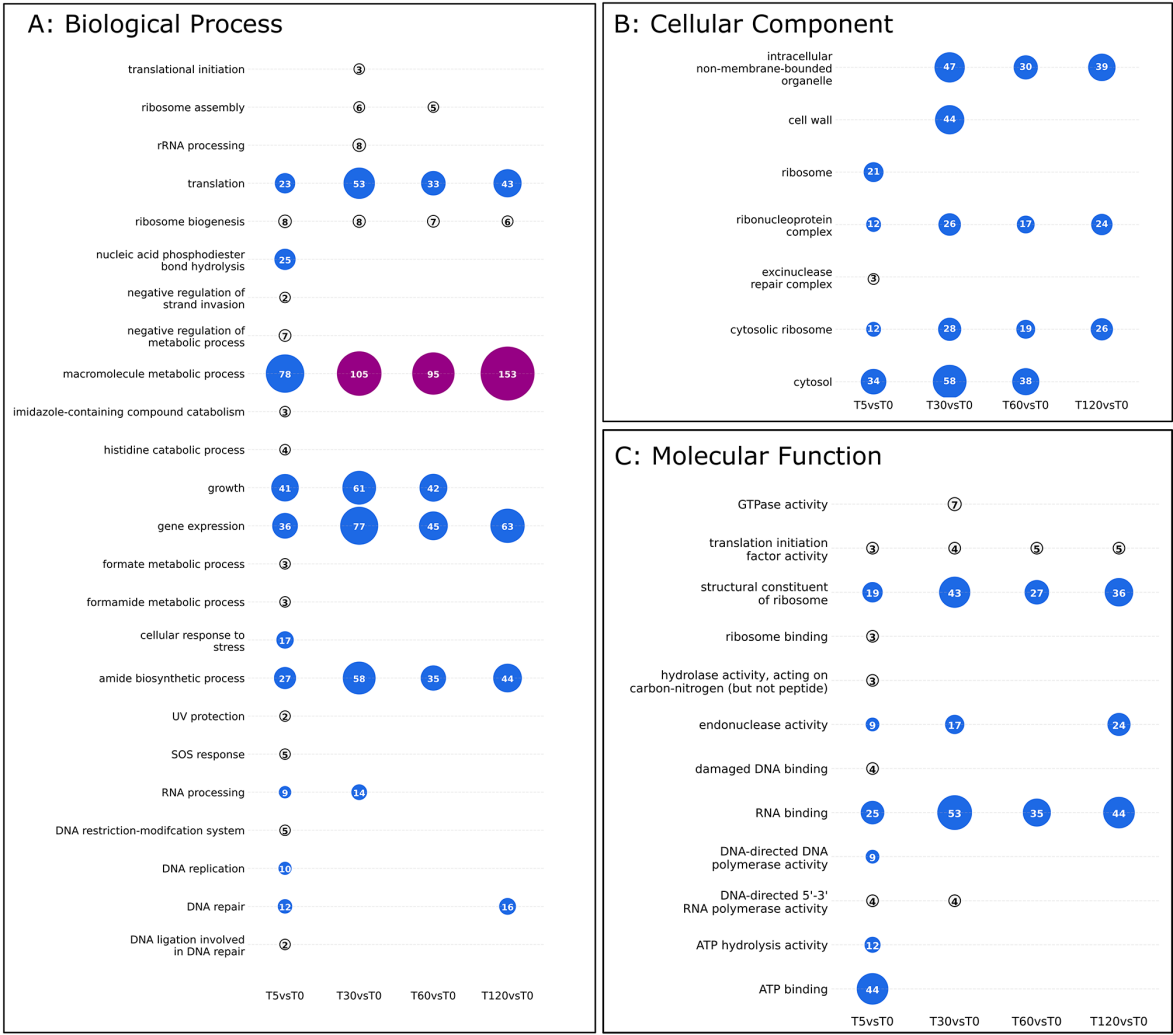
Enriched GO categories during time course of infection as compared to baseline by ontology. All categories indicated were up-regulated. The numbers inside the circles indicate the median gene count for each category. Categories were obtained as described in methods; the full list of terms in each category and individual gene counts for each term are provided in Supplementary Table S6. Only DE genes with absolute log_2_ fold change greater than or less than − 0.58 were included in the analysis to reflect a fold-change of 1.5

Enriched functional categories changed over the time course, reflecting a transcriptional response to the phases of phage infection (Figs. 5 and 6; Tables S7, S8, S9). At 5 min post-infection, phage WC1 was early in its latent period, beginning replication (Fig. 1A). Enriched terms, pathways, and genes in the host largely corresponded to host stress responses as well as DNA replication (Figs. 5 and 6; Tables S7, S8, S9). The enriched KEGG homologous recombination pathway included homologs of the SOS-response protein *RecA* (DVG_001424) and *RecC* (DVG80_03423) of the RecBCD pathway for double-stranded breaks [67]. *RecX* (DVG80_01423), the transcriptional mediator of *RecA*, was significantly up-regulated at 5 min as compared to baseline (log_2_FC: 1.69, *p*-value: 4.63E-7) as well. Other DNA repair mechanisms associated with the SOS-response were also enriched in both KEGG and GO, including activation of nucleotide excision repair, base excision repair and mismatch repair (Figs. 5 and 6) [67]. While KEGG pathways and GO terms also indicate significant up-regulation of DNA replication, which corresponds to phage takeover of host replication resources, there were also changes in host metabolism. These included significant up-regulation of ribosomal proteins necessary for mRNA translation and increases in histidine catabolism and glutamate biosynthesis (Figs. 5 and 6). Finally, DNA restriction-modification (RM) systems were enriched, which specifically corresponded to five up-regulated components of a Type I RM system (Fig. 6; Table S7).

At 30 min into the infection time course, the host response shifted significantly to increased transcriptional and translational activity in contrast to significant down-regulation of host metabolic pathways (Figs. 5 and 6). This corresponded to the substantial phage replication and protein synthesis occurring during the latent period of WC1 (Fig. 1A). Two KEGG pathways were up-regulated: RNA polymerase and ribosome, the latter of which consisted of 43 ribosomal proteins (Fig. 5). Enriched GO terms also indicate up-regulation of genes involved in gene expression, tRNAs, and ribosomal proteins as well as the formation of the ribosomal complex, translational initiation, and peptide biosynthesis (Fig. 6). Genes associated with the GO category macromolecule biosynthetic process at 30 min were largely ribosomal proteins, sigma factors, and translation elongation factors, but also included the LexA repressor, which is a transcriptional inhibitor of SOS-response related proteins [67]. A sequential comparison between 30 and 5 min (as opposed to baseline) showed significant down-regulation at 30 min of homologous recombination, mismatch repair, and nucleotide excision repair as well as many functions involved in DNA replication that had been enriched at the earlier time point (Figure S10; Table S10). Several KEGG pathways were down-regulated at 30 min as compared to the baseline, including those involved in carbon and energy metabolism, lipid metabolism, and branched-chain amino acid metabolism (Fig. 5). The down-regulated synthesis of secondary metabolites pathway included eight genes involved in the TCA cycle, five of which were part of the core down-regulated set (Table S4; Table S5).

After the end of the WC1 latent period, host transcriptional responses were highly similar to what was observed at 30 min. Direct comparison between 60 and 30 min showed no difference in functional enrichment, i.e., there were no significant KEGG pathways or GO terms when comparing 60 to 30 min (Fig. 5). When compared to baseline, up-regulated functional categories at 60 min were a subset of those at 30 min, predominantly related to transcription and translation (Figs. 5 and 6; Tables S7, S8, S9). Proteasomes were functionally enriched, with both the alpha and beta subunits, which are sufficient to construct the proteasome [68], as well as an associated ATPase up-regulated at 60 min. By 120 min post-infection, many of the functionally enriched host responses closely mirrored those observed at earlier stages of infection. The increase in transcriptional and translational activity from the baseline continued. However, DNA repair mechanisms including components of the SOS response were up-regulated as was endonuclease activity (Fig. 6; Table S7), similar to what was observed at 5 min, which corresponds to a potential second wave of phage infection (Fig. 1A). Oxidative phosphorylation was significantly suppressed at 120 min as compared to baseline, which was not observed at any other time point (Fig. 5).

## Discussion

In this study, we characterized WC-1, a new phage of *Rhodococcus erythropolis* RIA-643. While several *Rhodococcus* phage have previously been sequenced and annotated [20, 69], global patterns of gene expression during host infection have not yet been sufficiently explored. To the best of our knowledge, our study represents the first exploration of global gene expression following phage infection of a *Rhodococcus* host. We have described temporal dynamics of both phage and host transcriptomes, which may serve as a model not only for phage-host interactions in *R. erythropolis* RIA-643, but more generally for other strains and species in and beyond the genus.

WC1 followed an often reported pattern of phage transcriptional activation, expressing temporal classes of early, middle, and late genes [70–77]. These genes also clustered spatially within the genome, with early genes on the right arm, middle genes located more centrally, and late genes on the left arm. Central and left arm genes which could be annotated had regulatory functions and roles in DNA replication, while right arm genes included virion components and accessory factors for assembly. This genomic arrangement is syntenous with other cluster CA phage [20]. Rhodococcal phage have been shown to be polyvalent, with host ranges extending to other genera in some cases [15, 17, 78]. Highly similar phage of *Mycobacterium smegmatis* [22] as well as divergent phage of *Pseudomonas aeruginosa* [73] showed preservation of temporal activation patterns across synteny blocks. Taken together, these suggest that other cluster CA phage may follow patterns of expression similar to WC1, and may do so in multiple hosts. Furthermore, *M. smegmatis* phage displayed similar spatial arrangements and temporal expression patterns to WC1 including highly transcribed RNAs localized to non-coding regions [22, 23]. Three non-coding RNAs located at the far left, middle, and far right of the WC1 were among the most highly expressed transcripts at all time points except 5 min after infection. Similar ncRNAs at both the right and left extremes have been observed in Mycobacteriophages D29 and Kampy, while other cluster A Mycobacteriophage including L5, StarStuff, Redrock and SWU1 contain the right-hand ncRNA only [22, 23]. While the exact function of these noncoding regions is unknown, the region on the far right has been shown to be toxic to growth for both *M. smegmatis* and *E. coli*, and potentially essential for lytic growth [22].

Two main strategies for phage takeover of host cells have been previously described. The first involves widespread suppression, where the majority of host genes are down-regulated and host metabolism is shut down early in infection [71, 72, 79–82]. Alternatively, phage may exert more selective control, preserving the host’s regulatory mechanisms and upregulating more genes than inhibiting to create an environment most conducive for virion production [24, 74, 77, 83, 84]. Phage WC1 infection generated transcriptional profiles more congruent with the latter strategy, with close to equal proportions of up and down regulated host genes (both non-significant and DE), and a core set of DE genes as well as enriched functional categories maintained throughout the infection time course. Unique subsets of differentially expressed host genes also occurred at various time points and only 13% of host genes were DE during infection overall, which further supports more precise phage control of host transcription.

GO terms and KEGG pathways related to transcription and especially translation were significantly enriched from 5 min post-infection onward. During *Acinetobacter baumanii* infection by phage Abp1, phage early genes were associated with positive regulation of host gene expression [84], and similarly, early genes of phage JD032 were implicated in takeover of host cellular machinery in *Clostridium difficile* [72]. While the majority of WC1’s early genes are of unknown function, this early and consistent up-regulation of cellular machinery suggests that they may assist phage in commandeering host resources. Annotated early genes included an ArdA-like anti-restriction protein, while at the same time the host up-regulated a Type 1 restriction-modification system. ArdA proteins have been shown to be effective at inhibiting both the restriction and modification activities of Type I systems. The early host transcriptional profile also included activation of stress responses, including the SOS response, which has previously been shown to be inducible by phage [85]. We observed down-regulation of these stress responses and DNA repair mechanisms at 30 and 60 min, and then their return at 120 min as a putative new cycle of infection of remaining host cells was beginning.

A subset of genes involved in the TCA cycle and glycoxylate cycles were part of the down-regulated core set, and significant overall suppression of host carbon, carbohydrate, lipid, and amino acid metabolism began at 30 min post-infection. WC1 middle genes, which reached peak expression at this time point in the middle of the latent period, were largely involved in phage genome replication. Howard-Varona et al. observed a shift to the glycoxylate shunt for energy production upon *Pseudoalteromonas* infection with phage HP1, but not with phage HP2 [86]. Large-scale replication of phage WC1 did not appear to elicit a shift to the glycoxylate cycle, but rather a more general decrease in carbon metabolism and cellular energy production. This is further evidenced by significant down-regulation of oxidative phosphorylation later in the infection cycle. Similar mid to late stage deactivation in the TCA cycle and oxidative phosphorylation has been observed in infection by other phages [71, 82, 87]. The host transcriptional program continued at 60 and 120 min post-infection with few changes. Phage WC1 late genes, which peaked at 60 min and sustained high levels of expression at 120 min, were virion structural components and assembly factors.

The common core of DE host genes reflected the specific nature and regulation of host transcription during infection. Many of the core host genes which were up-regulated at all time points were regulatory including transcription factors as well as two small non-coding RNAs, F6 and the ydaO-yuaA riboswitch. In *M. tuberculosis*, the F6 sRNA is activated by oxidative and acid stress and leads to slow growth [52], while in *M. smegmatis*, deletion of F6 prevented cells from entering dormancy and down-regulated the resuscitation promoting factor RpfE2 [53]. Rpfs have been shown to promote cell growth and revitalize dormant cells in *Micrococcus luteus* [88], *Mycobacterium tuberculosis* [89, 90], and *Rhodococcus marinonascens* [91] as well as serving as essential factors for biofilm formation in *Mycobacterium smegmatis* [92]. During WC1 infection, both F6 and four rpf genes were significantly up-regulated at all time points, however, the RIA-643 homolog of *rpfE* was not one of these. Increased expression of F6 may be in response to stress, while increased expression of rpf genes may be driven by phage in order to prevent host cells from entering dormancy as a strategy to evade predation [93].

The core set of host genes also reflected potential changes in metal ion homeostasis, especially with respect to iron and manganese. In order to maintain iron homeostasis, bacterial cells employ high affinity transporters for import, ferritin and bacterioferritin intracellular storage systems, efflux transporters, and regulation of proteins that require iron cofactors in response to iron availability [94]. Manganese and iron have interrelated roles, with manganese able to substitute for iron as a cofactor during oxidative stress [95], and similar metal ion binding transcriptional regulators [94, 96]. A comparative study of *P. aeruginosa* phage determined that phage PAK_P4 up-regulated genes related to iron acquisition and transport, and that this was not part of a more general host stress response [73]. As hypothesized by Blasdel et al., this could have been either to provide iron intracellularly to be used as enzymatic cofactors, or to facilitate phage adsorption via tail fiber iron ions binding to siderophore receptors [73], as described by the “Ferrojan Horse Hypothesis” [97]. In contrast, phage SWU infection of *M. smegmatis* led to transcriptional suppression of genes involved in siderophore synthesis [24]. Up-regulation of bacterioferritin was observed during *P. aeruginosa* infection with phi KZ [98]. During WC1 infection, we saw increased expression of metal ion ABC transporters including those mediated by siderophores as well as decreased expression of metal ion exporters and bacterioferritin. This was coupled with low expression throughout infection of a homolog to the iron-binding furAB repressor, and non-DE suppression of expression of a putative iron-binding ideR repressor. First described in *E. coli*, fur family repressors bind iron in times of sufficient supply to suppress transcription of genes related to iron import [99]. The ideR repressor also binds iron and regulates responses to oxidative stress in *M. tuberculosis*, and has also been shown to be present in other strains of *R. erythropolis* as well as active in *R. equi* [100]. The combination of increased import and decreased export and sequestration with a lack of transcriptional repression which would be indicative of high iron conditions suggests selective phage-mediated control to increase intracellular iron concentrations to facilitate virion replication and assembly. We also note the presence of a phage gene encoding a Cas exonuclease, however no identifiable CRISPR systems are present in the host genome, a result reported for other *Rhodococcus* species [101, 102].

There are apparent limitations of the current study, including the large number of phage and host gene products of unknown function. As genomic and metagenomic sequencing efforts continue *en masse*, future characterization of these proteins may become possible. Phage WC1 bears strong identity to known temperate phage, and contains integrase and excise, hence indicating that it is capable of lysogeny. We observed that these genes had constitutively low expression during the infection time course. In order to search for alignments between the phage and host genomes, the phage genome was processed into non-overlapping 1000 bp pieces. Analysis with blastn showed no alignments meeting default non-stringent BLAST thresholds (E-value cutoff of 10). However, in the current study, we do not know what proportion of host cells may be lysogens, and further to that, how many may be uninfected. Finally, further exploration with downstream ‘omics, namely proteomics and metabolomics, will be necessary to confirm these results as well as shed further light on interactions beyond the transcriptional level.

Here, we have shed light on previously undescribed transcriptional dynamics during phage infection of a *Rhodococcus* host, using the novel phage WC1. Given the high level of similarity between phage of cluster CA, and polyvalence of *Rhodococcus* phage, results from this system may have broader applicability to predator-prey dynamics in a range of environmentally, industrially, and biomedically important bacterial hosts. Further elucidation of the interplay between *Rhodococcus* phage and their hosts will be essential for the advancement of phage-mediated biocontrol strategies.

## Conclusions

The *Rhodococcus* genus is well recognized for its ability to synthesize valuable compounds, particularly steroids, as well as its capacity to degrade a wide range of harmful environmental pollutants. A detailed understanding of these phage-host interactions and gene expression is not only essential for understanding the ecology of this important genus, but will also facilitate development of phage-mediated strategies for bioremediation as well as biocontrol in industrial processes and biomedical applications. Given the current lack of detailed global gene expression studies on any *Rhodococcus* species, our study addresses a pressing need to identify tools and genes, such as *F6* and *rpf*, that can enhance the capacity of *Rhodococcus* species for bioremediation, biosynthesis and pathogen control. While host gene expression declined over the course of infection, our results indicate that phage may exert more selective control, preserving the host’s regulatory mechanisms to create an environment conducive for virion production.

### Electronic supplementary material

Below is the link to the electronic supplementary material.

Supplementary Material 1

## Data Availability

The complete genome sequence of WC1 was deposited in Genbank and is available via accession number MZ402608. RNA-Sequencing data can be accessed via NCBI’s Short Read Archive at project accession number SRP154435.

## Abbreviations

SEA-PHAGES: Science Education Alliance Phage Hunters Advancing Genomics and Evolutionary Science
WC1: Winter Compost 1
DE: Differential expression/Differentially Expressed
