# Supplementary material for: Transcriptional dynamics during Rhodococcus erythropolis infection with phage WC1
Source: BMC Microbiol. 2024 Apr 1;24:107. doi: 10.1186/s12866-024-03241-4 (PMC10986025; doi:10.1186/s12866-024-03241-4)
Supplement: Supplementary file 1 — Supplementary Material 1 [file 12866_2024_3241_MOESM1_ESM.docx]

**Supplementary Material**

**
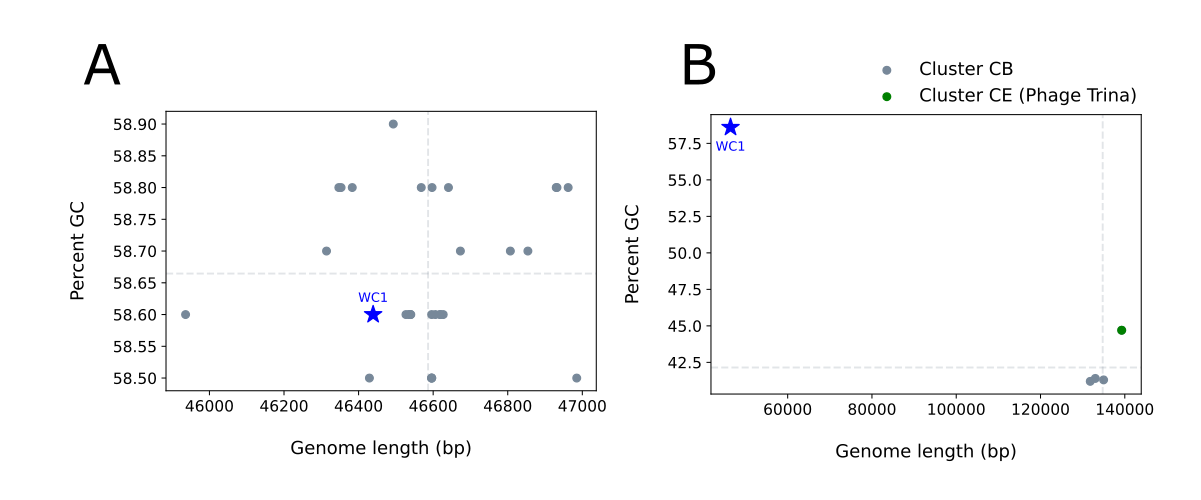
**

**Figure S1:** Genome lengths and GC content for previously identified R. erythropolis RIA-643 phage versus WC1. (A) shows data for phage in cluster CA, while (B) shows for comparison phage from clusters CB and CE.


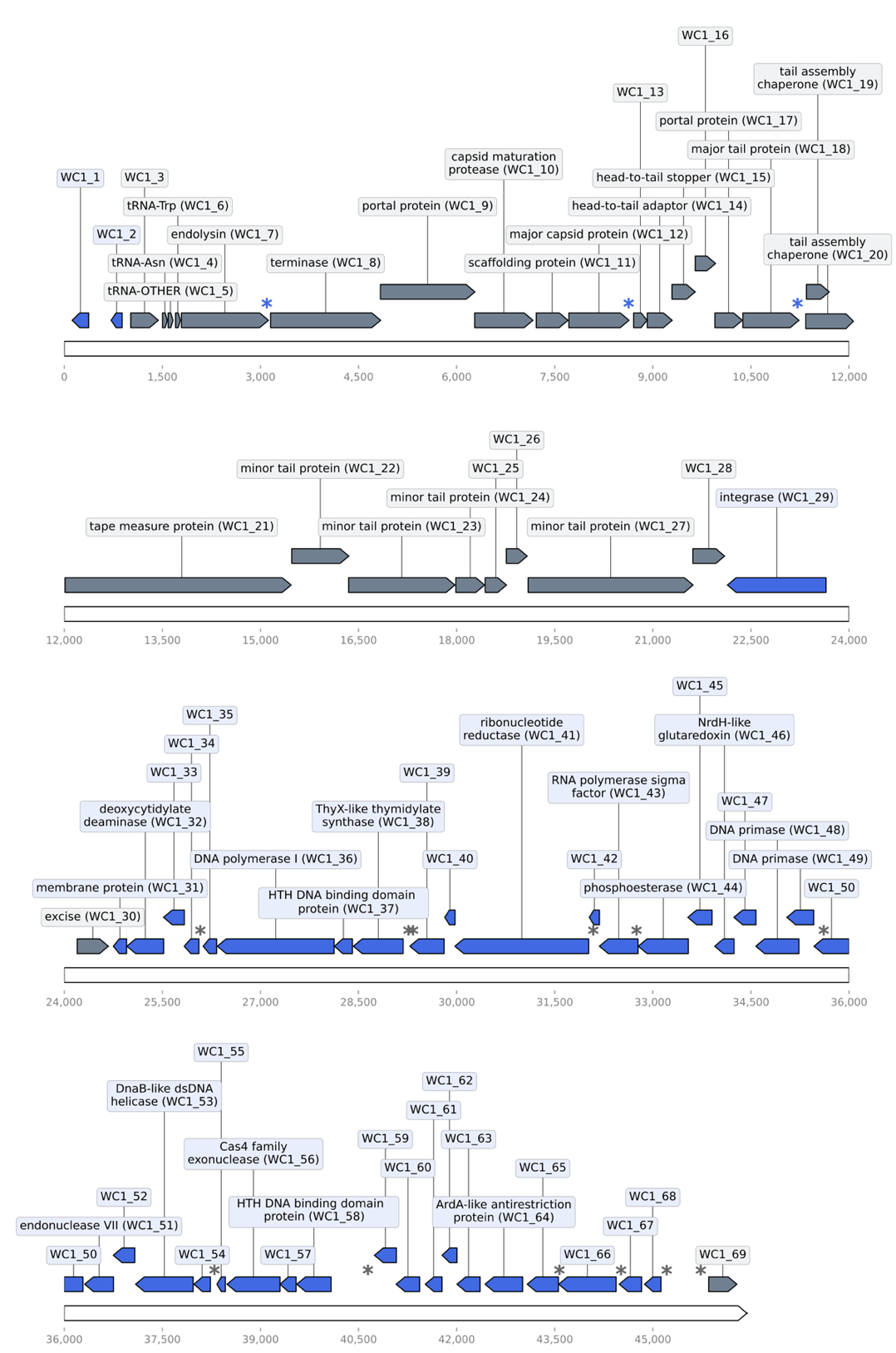


**Figure S2:** Genome structure of WC1. Protein-coding genes in forward orientation are shown in grey, and those in reverse are shown in blue. Stars indicate 13mer regulatory sequences: blue stars are reverse complemented sequences. Functional annotations where available and gene numbers are included in labels.


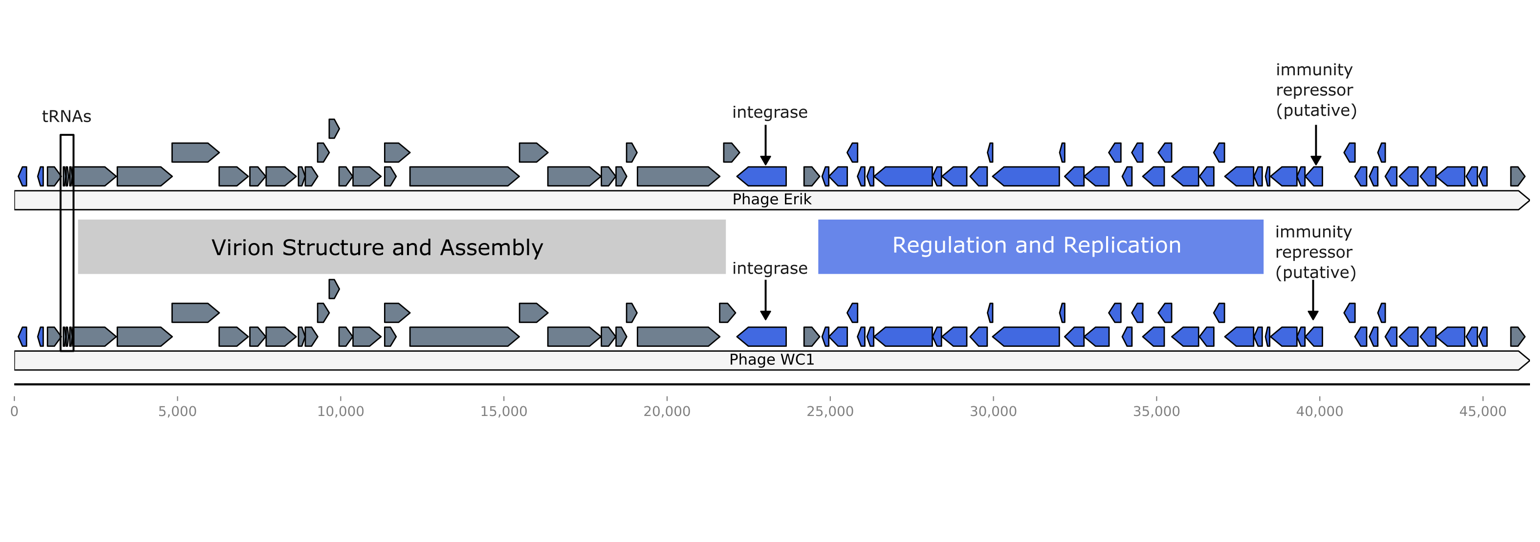


**Figure S3.** Genomic arrangement of Phage WC1 and Phage Erik. Erik was chosen as a representative phage for cluster CA phage of R. erythropolis RIA-643. The terms “Virion Structure and Assembly” and “Regulation and Replication” are the descriptors used in (20)to characterize regions of CA phage genomes.

**
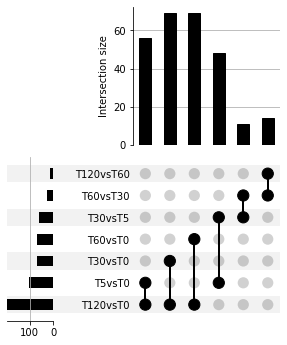
**

**Figure S4:** DE set composition and intersection between selected comparisons for phage genes. DE genes were identified as those having |FC| > 1.5 and p<0.05 in pairwise Wald tests. The height of each vertical bar shows the size of the intersection between the two comparisons indicated by dots. The length of each horizontal bar shows how many DE genes were in each individual comparison.


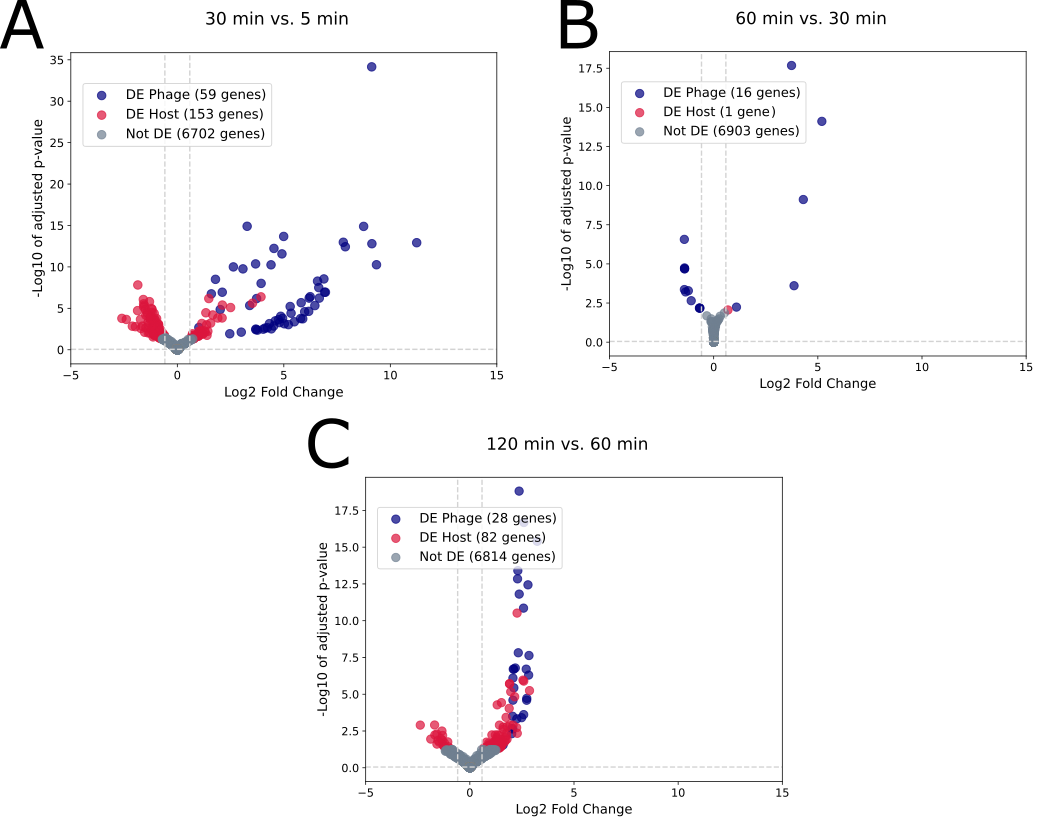


**Figure S5.** Differentially expressed (DE) genes over the time course of infection. Each point on the volcano plot is based on 2 replicates. Vertical lines indicate Log_2_ Fold Change of 0.58 and -0.58, which corresponds to a fold change of 1.5. The horizontal line indicates p of 0.05.

**
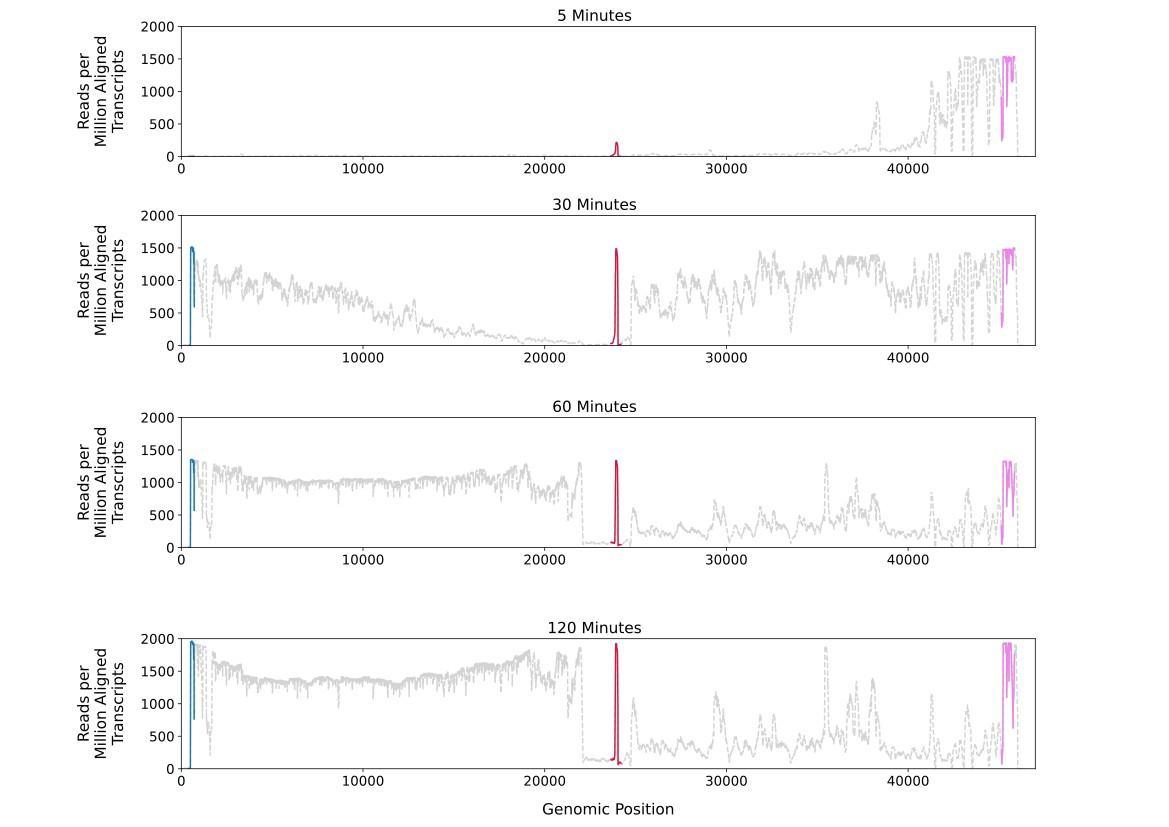
**

**Figure S6:** Overall genome coverage for reads mapped at each time point. Non-coding regions are indicated in color. Values on the y-axis represent the average of two normalized values for two replicates.

**
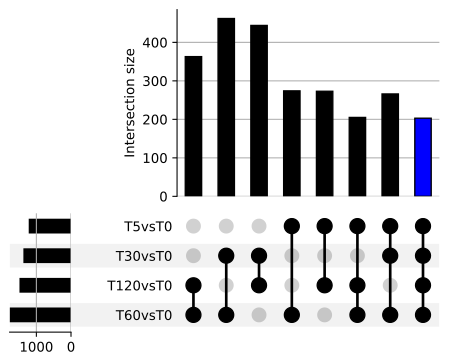
**

**Figure S7.** DE set composition and intersection between selected comparisons for host genes with baseline, with the blue bar showing the complete overlap of 203 genes between all sets. DE genes were identified as those having |FC| > 1.5 and p<0.05 in pairwise Wald tests. The height of each vertical bar shows the size of the intersection between the two comparisons indicated by dots. The length of each horizontal bar shows how many DE genes were in each individual comparison.


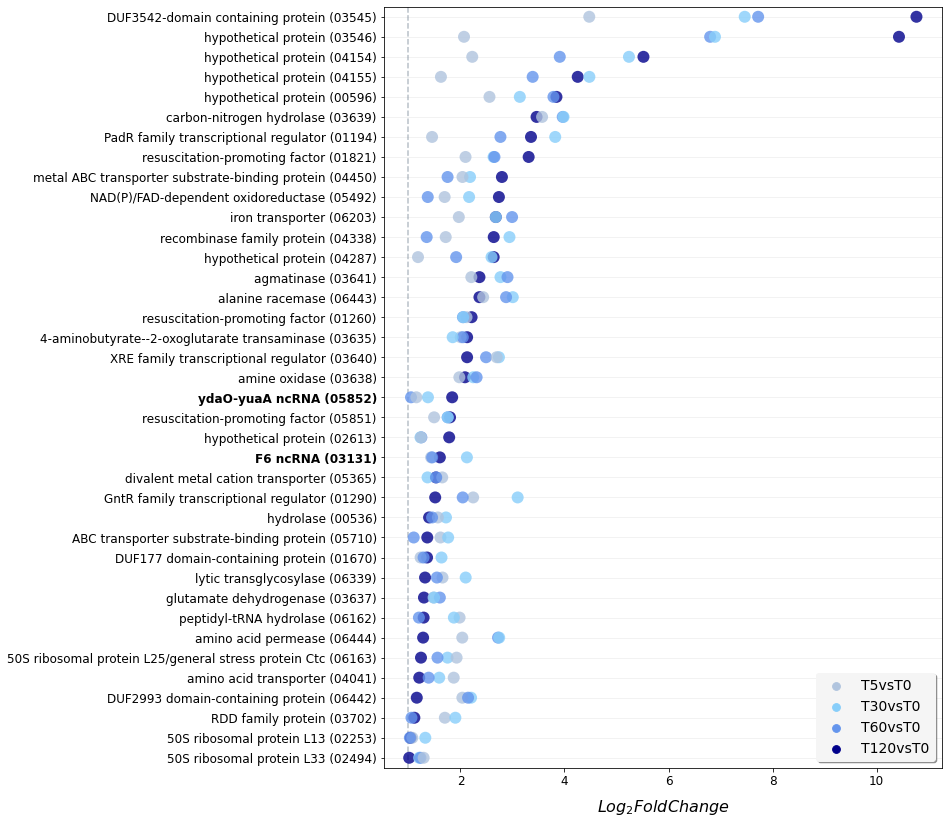


**Figure S8.** Core subset of 38 universally up-regulated DE genes and ncRNAs (in bold) with FC > 2 at all infection time points versus baseline (time 0). The feature identifier in RIA-643 is provided in parentheses after the description. The full list of up-regulated DE genes in the core set with |FC| > 1.5 is provided in Table S4.


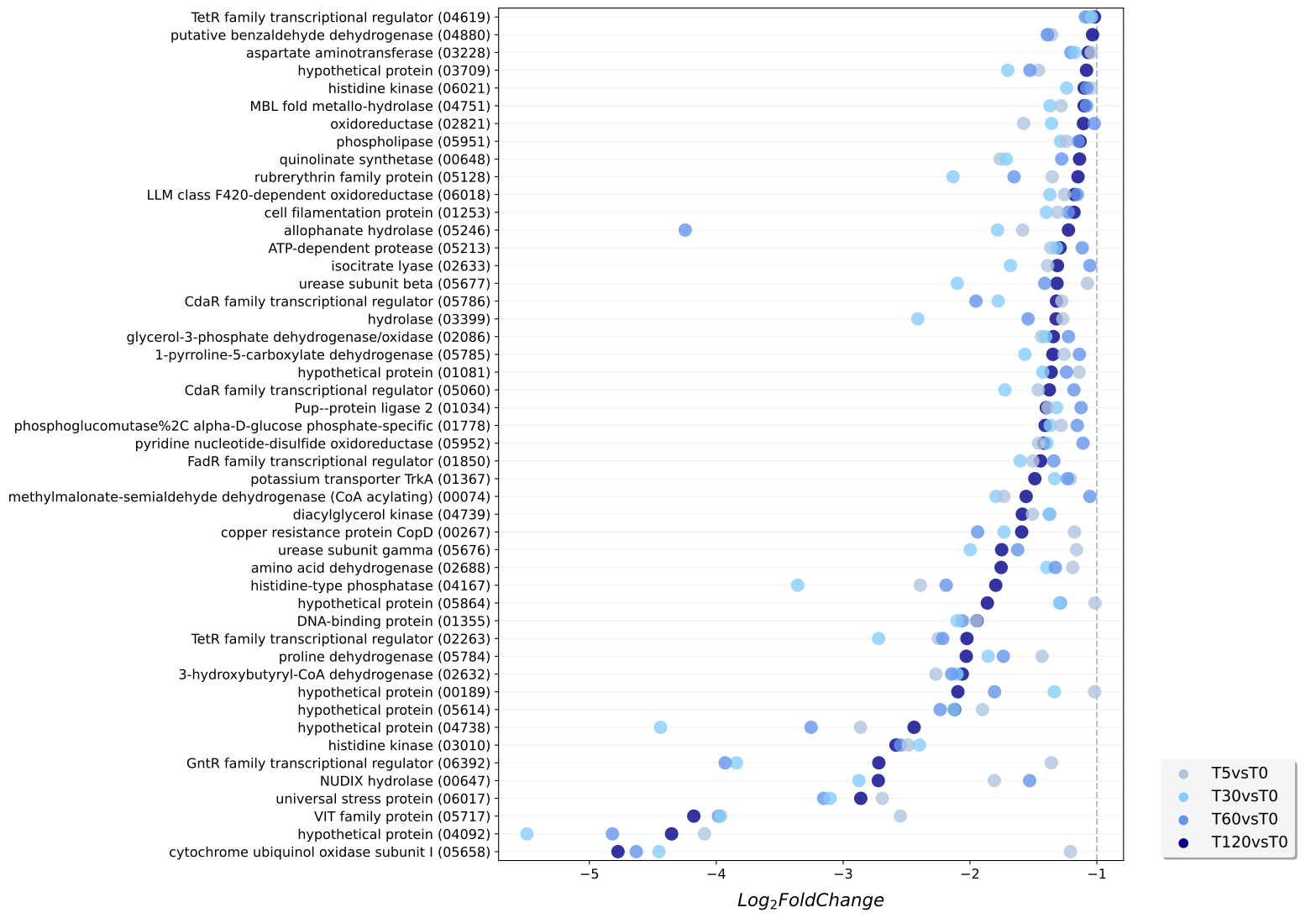


**Figure S9.** Core subset of 48 universally down-regulated DE genes with FC > 2 at all infection time points versus baseline (time 0). The feature identifier in RIA-643 is provided in parentheses after the description. The full list of up-regulated DE genes in the core set with |FC |> 1.5 is provided in Table S4.


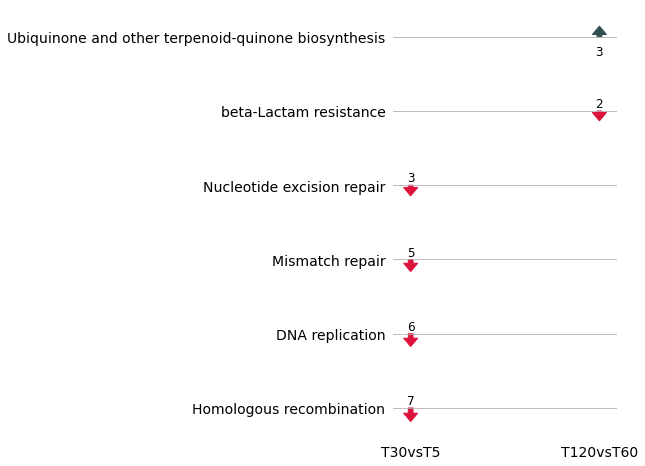


**Figure S10.** Enriched KEGG pathways during time course of infection for time points compared sequentially. The direction of the arrows indicate if the DE genes were up-regulated or down-regulated, and the numeric values indicate the number of genes. Only DE genes with absolute log_2_ fold change greater than or less than -0.58 were included in the analysis to reflect a fold-change of 1.5. T60vsT30 is not included because there were no significant pathways.

**Table S1**. Phage used for comparative analysis of WC-1 genome. Accession numbers and cluster memberships were obtained from phagedb at (https://phagesdb.org/clusters/).

| **Phage name** | **Accession number** | **Cluster** |
| --- | --- | --- |
| Alatin | MF324905 | CA |
| Alpacados | MH271291 | CA |
| AngryOrchard | KY549153 | CA |
| AppleCloud | MF324903 | CA |
| Belenaria | MK524495 | CA |
| BobbyDazzler | KY549154 | CA |
| Bonanza | MF537628 | CA |
| Bradshaw | MH271293 | CA |
| Bryce | MH271294 | CA |
| CosmicSans | KT372002 | CA |
| Dinger | MN945902 | CA |
| Erik | MH271297 | CA |
| Espica | MK524487 | CA |
| Gollum | MH271299 | CA |
| Harlequin | KX611788 | CA |
| Hiro | MF324898 | CA |
| Jester | MF373842 | CA |
| Krishelle | MF324902 | CA |
| Lillie | KT990218 | CA |
| Naiad | MF324901 | CA |
| Nancinator | MH271306 | CA |
| Natosaleda | KX550082 | CA |
| Partridge | KX712237 | CA |
| Phrankenstein | MH271309 | CA |
| Rasputin | MH271311 | CA |
| RexFury | MF324904 | CA |
| Rhodalysa | KT375356 | CA |
| StCroix | MF324900 | CA |
| Swann | MH271314 | CA |
| Takoda | MH271315 | CA |
| TWAMP | KT959213 | CA |
| UhSalsa | MH271319 | CA |
| Yogi | KX712236 | CA |
| Yoncess | MF189179 | CA |
| Grayson | MH153812 | CB |
| Peregrin | MH153807 | CB |
| Weasels2 | KX774321 | CB |
| Trina | MF668286 | CE |

**Table S2.** Read counts for RNA-seq libraries. Number and percentages of unmapped reads determined based on alignment with HISAT2 to the combined phage and host genomes.

| **Sample** | **Total reads** | **Unmapped Reads** | **Percent mapped** |
| --- | --- | --- | --- |
| **T0-1** | 6360952 | 156538 | 97.54% |
| **T0-2** | 3862912 | 186933 | 95.16% |
| **T5-1** | 7552000 | 156930 | 97.92% |
| **T5-2** | 3914540 | 125504 | 96.79% |
| **T30-1** | 5144798 | 132338 | 97.43% |
| **T30-2** | 5436524 | 163131 | 97.00% |
| **T60-1** | 5020254 | 255234 | 94.92% |
| **T60-2** | 7153734 | 152833 | 97.86% |
| **T120-1** | 3051712 | 161191 | 94.72% |
| **T120-2** | 6151346 | 146705 | 97.62% |

**Table S3.** Reads mapping to phage WC1 genome at time 0 (T0).

| **Replicate** | **Count Phage Reads Assigned** | **Total Reads Assigned** | **Percentage phage reads of total** |
| --- | --- | --- | --- |
| T0-1 | 654 | 5805222 | 0.011% |
| T0-2 | 512 | 3428880 | 0.015% |

**Table S4.**  Core subset of universally DE genes with |FC |> 1.5 at all infection time points versus baseline (time 0). P-values were adjusted using the Benjamini-Hochberg method.

| **Gene** | **Description** | **5 minutes** | | **30 minutes** | | **60 minutes** | | **120 minutes** | |
| --- | --- | --- | --- | --- | --- | --- | --- | --- | --- |
|  |  | **log_2_FC** | **p-value (BH)** | **log_2_FC** | **p-value (BH)** | **log_2_FC** | **p-value (BH)** | **log_2_FC** | **p-value (BH)** |
| DVG80_03545 | hypothetical protein | 4.48 | 4.65E-08 | 7.47 | 2.72E-21 | 7.72 | 9.26E-23 | 10.77 | 1.87E-44 |
| DVG80_03546 | hypothetical protein | 2.07 | 5.14E-04 | 6.89 | 3.02E-18 | 6.80 | 1.13E-17 | 10.43 | 6.99E-42 |
| DVG80_04154 | hypothetical protein | 2.23 | 1.33E-05 | 5.24 | 1.37E-18 | 3.91 | 4.45E-11 | 5.52 | 5.71E-20 |
| DVG80_04155 | hypothetical protein | 1.62 | 1.82E-04 | 4.48 | 6.86E-21 | 3.39 | 3.97E-13 | 4.25 | 6.55E-20 |
| DVG80_00596 | hypothetical protein | 2.56 | 8.58E-06 | 3.14 | 7.47E-07 | 3.79 | 1.41E-08 | 3.85 | 1.42E-09 |
| DVG80_01195 | hypothetical protein | 0.96 | 3.12E-02 | 4.19 | 4.59E-12 | 2.06 | 7.84E-05 | 3.62 | 7.36E-10 |
| DVG80_03639 | carbon-nitrogen hydrolase | 3.57 | 3.04E-24 | 3.98 | 1.97E-29 | 3.97 | 1.22E-29 | 3.46 | 2.94E-23 |
| DVG80_01194 | PadR family transcriptional regulator | 1.45 | 1.03E-03 | 3.82 | 1.06E-14 | 2.77 | 5.26E-09 | 3.36 | 7.81E-12 |
| DVG80_01821 | resuscitation-promoting factor | 2.10 | 9.09E-12 | 2.64 | 1.07E-17 | 2.65 | 1.38E-17 | 3.31 | 4.90E-26 |
| DVG80_04450 | metal ABC transporter substrate-binding protein | 2.04 | 3.08E-04 | 2.18 | 1.59E-04 | 1.75 | 1.19E-03 | 2.80 | 1.92E-05 |
| DVG80_05492 | NAD(P)/FAD-dependent oxidoreductase | 1.70 | 9.67E-06 | 2.17 | 7.80E-09 | 1.37 | 1.87E-04 | 2.74 | 8.10E-11 |
| DVG80_06203 | iron transporter | 1.97 | 1.66E-06 | 2.68 | 1.64E-10 | 2.99 | 4.04E-12 | 2.68 | 5.47E-08 |
| DVG80_04338 | recombinase family protein | 1.72 | 2.47E-05 | 2.94 | 1.70E-12 | 1.35 | 4.56E-04 | 2.64 | 4.94E-09 |
| DVG80_04287 | hypothetical protein | 1.18 | 1.69E-02 | 2.60 | 5.91E-05 | 1.92 | 9.26E-04 | 2.64 | 1.80E-04 |
| DVG80_06010 | hypothetical protein | 1.28 | 8.21E-03 | 0.95 | 3.18E-02 | 0.86 | 4.39E-02 | 2.63 | 1.36E-05 |
| DVG80_02595 | aromatic compound degradation protein PaaI | 0.95 | 9.42E-03 | 1.13 | 1.67E-03 | 0.79 | 2.95E-02 | 2.38 | 2.54E-09 |
| DVG80_03641 | agmatinase | 2.21 | 8.55E-08 | 2.77 | 8.24E-11 | 2.90 | 2.41E-11 | 2.36 | 5.62E-08 |
| DVG80_06443 | alanine racemase | 2.44 | 1.05E-14 | 3.01 | 1.15E-20 | 2.88 | 4.76E-19 | 2.36 | 1.07E-12 |
| DVG80_01260 | resuscitation-promoting factor | 2.12 | 1.14E-08 | 2.05 | 2.46E-08 | 2.05 | 4.98E-08 | 2.21 | 6.63E-07 |
| DVG80_03635 | 4-aminobutyrate--2-oxoglutarate transaminase | 2.00 | 1.65E-07 | 1.85 | 8.18E-07 | 2.05 | 9.38E-08 | 2.13 | 1.05E-07 |
| DVG80_03640 | XRE family transcriptional regulator | 2.70 | 1.05E-14 | 2.74 | 8.11E-15 | 2.49 | 2.22E-12 | 2.13 | 4.02E-08 |
| DVG80_03638 | amine oxidase | 1.98 | 2.02E-12 | 2.25 | 5.84E-16 | 2.31 | 1.76E-16 | 2.09 | 4.29E-13 |
| DVG80_05852 | ydaO-yuaA | 1.15 | 1.06E-04 | 1.37 | 1.45E-06 | 1.05 | 2.43E-04 | 1.84 | 4.26E-10 |
| DVG80_05851 | resuscitation-promoting factor | 1.49 | 9.84E-07 | 1.76 | 1.79E-09 | 1.75 | 1.91E-09 | 1.80 | 3.95E-09 |
| **Gene** | **Description** | **5 minutes** | | **30 minutes** | | **60 minutes** | | **120 minutes** | |
|  |  | **log_2_FC** | **p-value (BH)** | **log_2_FC** | **p-value (BH)** | **log_2_FC** | **p-value (BH)** | **log_2_FC** | **p-value (BH)** |
| DVG80_02613 | hypothetical protein | 1.24 | 2.39E-04 | 1.23 | 1.55E-04 | 1.25 | 9.85E-05 | 1.78 | 1.21E-06 |
| DVG80_04723 | hypothetical protein | 1.12 | 1.86E-02 | 0.97 | 3.04E-02 | 1.47 | 2.40E-03 | 1.72 | 5.97E-03 |
| DVG80_03542 | DsbA family oxidoreductase | 1.53 | 1.36E-06 | 1.22 | 8.74E-05 | 0.94 | 2.60E-03 | 1.65 | 7.51E-06 |
| DVG80_03131 | F6 ncRNA | 1.43 | 2.33E-03 | 2.12 | 1.36E-05 | 1.46 | 1.28E-03 | 1.60 | 3.54E-03 |
| DVG80_05365 | divalent metal cation transporter | 1.65 | 1.45E-03 | 1.37 | 4.44E-03 | 1.53 | 1.93E-03 | 1.53 | 8.89E-03 |
| DVG80_01290 | GntR family transcriptional regulator | 2.24 | 3.30E-08 | 3.10 | 3.77E-13 | 2.04 | 7.35E-07 | 1.51 | 2.22E-03 |
| DVG80_03824 | ABC transporter substrate-binding protein | 0.80 | 3.99E-02 | 1.37 | 6.89E-04 | 1.14 | 3.67E-03 | 1.46 | 1.39E-03 |
| DVG80_00536 | hydrolase | 1.57 | 2.28E-06 | 1.72 | 5.81E-08 | 1.45 | 3.81E-06 | 1.40 | 3.09E-05 |
| DVG80_05839 | LLM class flavin-dependent oxidoreductase | 0.78 | 4.12E-02 | 1.49 | 1.71E-04 | 0.87 | 2.25E-02 | 1.39 | 2.82E-03 |
| DVG80_04554 | inositol-3-phosphate synthase | 0.93 | 8.69E-05 | 1.47 | 6.34E-11 | 1.46 | 6.77E-11 | 1.39 | 8.09E-09 |
| DVG80_05710 | ABC transporter substrate-binding protein | 1.62 | 8.43E-04 | 1.76 | 1.96E-04 | 1.10 | 9.97E-03 | 1.36 | 1.02E-02 |
| DVG80_01012 | hypothetical protein | 0.73 | 4.54E-02 | 0.87 | 2.20E-02 | 1.61 | 2.40E-05 | 1.36 | 3.24E-03 |
| DVG80_01670 | hypothetical protein | 1.23 | 4.24E-04 | 1.63 | 1.06E-06 | 1.29 | 9.17E-05 | 1.36 | 1.18E-04 |
| DVG80_02724 | ABC transporter ATP-binding protein | 1.12 | 5.55E-03 | 1.55 | 9.09E-05 | 0.81 | 3.31E-02 | 1.34 | 2.13E-03 |
| DVG80_06339 | lytic transglycosylase | 1.65 | 1.87E-08 | 2.10 | 1.53E-13 | 1.55 | 4.61E-08 | 1.32 | 1.62E-05 |
| DVG80_03637 | glutamate dehydrogenase | 1.48 | 9.53E-10 | 1.49 | 1.64E-10 | 1.60 | 4.98E-12 | 1.30 | 1.16E-07 |
| DVG80_06162 | peptidyl-tRNA hydrolase | 1.98 | 4.67E-06 | 1.87 | 1.03E-05 | 1.20 | 2.51E-03 | 1.29 | 5.29E-03 |
| DVG80_06444 | amino acid permease | 2.03 | 8.77E-06 | 2.74 | 1.26E-08 | 2.72 | 2.81E-08 | 1.28 | 6.90E-03 |
| DVG80_00882 | translation initiation factor IF-3 | 1.15 | 2.97E-04 | 1.36 | 8.61E-06 | 0.96 | 1.75E-03 | 1.24 | 9.46E-05 |
| DVG80_06163 | 50S ribosomal protein L25/general stress protein Ctc | 1.93 | 3.46E-06 | 1.75 | 1.40E-05 | 1.56 | 9.02E-05 | 1.24 | 3.44E-03 |
| DVG80_00217 | amidase | 0.99 | 8.16E-04 | 1.21 | 2.75E-05 | 0.83 | 4.99E-03 | 1.21 | 2.20E-04 |
| DVG80_04452 | metal ABC transporter permease | 0.88 | 4.46E-02 | 1.08 | 1.59E-02 | 0.89 | 3.48E-02 | 1.21 | 2.17E-02 |
| DVG80_01138 | ABC transporter ATP-binding protein | 0.88 | 5.99E-03 | 1.23 | 9.34E-05 | 1.15 | 2.31E-04 | 1.21 | 3.18E-04 |
| DVG80_04041 | amino acid transporter | 1.87 | 7.28E-05 | 1.59 | 3.74E-04 | 1.39 | 1.41E-03 | 1.21 | 1.18E-02 |
| DVG80_06075 | gamma-aminobutyraldehyde dehydrogenase | 0.77 | 1.65E-02 | 1.79 | 1.80E-08 | 1.96 | 1.03E-09 | 1.19 | 6.34E-04 |
|  |  |  | |  | |  | |  | |
|  |  |  | |  | |  | |  | |
| **Gene** | **Description** | **5 minutes** | | **30 minutes** | | **60 minutes** | | **120 minutes** | |
|  |  | **log_2_FC** | **p-value (BH)** | **log_2_FC** | **p-value (BH)** | **log_2_FC** | **p-value (BH)** | **log_2_FC** | **p-value (BH)** |
| DVG80_06442 | hypothetical protein | 2.04 | 1.17E-11 | 2.20 | 1.10E-13 | 2.15 | 9.01E-13 | 1.16 | 7.92E-04 |
| DVG80_01389 | peroxiredoxin | 0.59 | 3.75E-02 | 1.04 | 2.47E-04 | 1.47 | 7.01E-08 | 1.14 | 1.12E-04 |
| DVG80_04495 | hypothetical protein | 2.62 | 1.23E-24 | 1.54 | 2.22E-09 | 0.85 | 1.71E-03 | 1.13 | 1.52E-04 |
| DVG80_03702 | RDD family protein | 1.70 | 2.17E-06 | 1.90 | 4.29E-08 | 1.06 | 1.84E-03 | 1.11 | 3.83E-03 |
| DVG80_00909 | hypothetical protein | 1.17 | 9.60E-04 | 1.51 | 1.03E-05 | 0.90 | 8.07E-03 | 1.09 | 3.27E-03 |
| DVG80_03957 | AraC family transcriptional regulator | 1.25 | 6.07E-03 | 1.72 | 1.71E-04 | 0.95 | 2.05E-02 | 1.09 | 2.34E-02 |
| DVG80_03634 | NAD-dependent succinate-semialdehyde dehydrogenase | 1.46 | 1.01E-04 | 1.01 | 4.73E-03 | 0.88 | 1.22E-02 | 1.08 | 4.38E-03 |
| DVG80_02253 | 50S ribosomal protein L13 | 1.07 | 1.10E-03 | 1.32 | 3.35E-05 | 1.03 | 1.20E-03 | 1.04 | 2.13E-03 |
| DVG80_04087 | membrane protein | 1.13 | 5.21E-03 | 0.97 | 1.29E-02 | 0.84 | 2.71E-02 | 1.03 | 2.61E-02 |
| DVG80_04555 | PadR family transcriptional regulator | 0.89 | 8.10E-04 | 1.32 | 2.55E-07 | 1.24 | 1.04E-06 | 1.01 | 3.10E-04 |
| DVG80_02494 | 50S ribosomal protein L33 | 1.29 | 4.95E-04 | 1.20 | 6.65E-04 | 1.23 | 4.07E-04 | 1.01 | 7.30E-03 |
| DVG80_00587 | hypothetical protein | 0.61 | 3.17E-02 | 0.94 | 1.09E-03 | 0.93 | 1.04E-03 | 0.95 | 2.00E-03 |
| DVG80_04376 | hypothetical protein | 1.12 | 6.05E-03 | 1.62 | 6.33E-05 | 1.03 | 7.00E-03 | 0.94 | 2.73E-02 |
| DVG80_00535 | ydaO-yuaA | 1.20 | 7.86E-04 | 1.15 | 8.78E-04 | 0.95 | 5.13E-03 | 0.92 | 1.30E-02 |
| DVG80_06055 | metallopeptidase | 0.79 | 1.60E-02 | 1.17 | 3.26E-04 | 1.30 | 5.39E-05 | 0.91 | 1.03E-02 |
| DVG80_00883 | 50S ribosomal protein L35 | 0.95 | 1.65E-03 | 1.03 | 5.83E-04 | 0.82 | 6.32E-03 | 0.90 | 5.42E-03 |
| DVG80_01975 | ATP-dependent helicase | 1.02 | 1.01E-03 | 0.74 | 1.95E-02 | 0.85 | 5.27E-03 | 0.90 | 6.61E-03 |
| DVG80_02444 | 30S ribosomal protein S12 | 0.70 | 2.89E-02 | 1.05 | 1.31E-03 | 0.79 | 1.67E-02 | 0.89 | 1.14E-02 |
| DVG80_02284 | 50S ribosomal protein L24 | 0.83 | 8.15E-03 | 1.12 | 3.36E-04 | 0.87 | 5.79E-03 | 0.88 | 8.67E-03 |
| DVG80_01668 | ribonuclease III | 0.83 | 5.32E-03 | 1.03 | 6.12E-04 | 0.70 | 2.40E-02 | 0.88 | 5.88E-03 |
| DVG80_06126 | resuscitation-promoting factor | 0.70 | 1.84E-02 | 1.14 | 1.21E-04 | 0.86 | 4.32E-03 | 0.85 | 9.11E-03 |
| DVG80_00114 | peptide chain release factor 1 | 1.03 | 6.34E-04 | 1.26 | 1.83E-05 | 0.99 | 7.66E-04 | 0.84 | 1.17E-02 |
| DVG80_01525 | polyribonucleotide nucleotidyltransferase | 0.83 | 4.73E-03 | 1.00 | 7.02E-04 | 0.88 | 2.73E-03 | 0.84 | 8.04E-03 |
| DVG80_02492 | 3-hydroxyacyl-ACP dehydratase | 1.26 | 5.70E-04 | 1.27 | 2.88E-04 | 0.88 | 1.10E-02 | 0.83 | 2.75E-02 |
|  |  |  | |  | |  | |  | |
| Gene | Description | 5 minutes | | 30 minutes | | 60 minutes | | 120 minutes | |
|  |  | log_2_FC | p-value (BH) | log_2_FC | p-value (BH) | log_2_FC | p-value (BH) | log_2_FC | p-value (BH) |
| DVG80_00885 | RNA methyltransferase | 1.18 | 1.35E-04 | 1.28 | 1.97E-05 | 0.89 | 3.50E-03 | 0.83 | 1.30E-02 |
| DVG80_02285 | 50S ribosomal protein L14 | 0.68 | 3.70E-02 | 1.16 | 4.62E-04 | 0.78 | 2.00E-02 | 0.82 | 2.26E-02 |
| DVG80_02252 | 30S ribosomal protein S9 | 0.75 | 1.99E-02 | 1.07 | 1.11E-03 | 0.79 | 1.71E-02 | 0.81 | 2.31E-02 |
| DVG80_02272 | translation initiation factor IF-1 | 0.70 | 2.17E-02 | 1.02 | 1.09E-03 | 0.90 | 3.67E-03 | 0.80 | 1.61E-02 |
| DVG80_02493 | acyl dehydratase | 1.14 | 1.02E-03 | 1.38 | 3.98E-05 | 0.97 | 3.63E-03 | 0.80 | 2.78E-02 |
| DVG80_01667 | formamidopyrimidine-DNA glycosylase | 1.14 | 6.67E-04 | 1.07 | 1.04E-03 | 0.83 | 1.05E-02 | 0.78 | 2.91E-02 |
| DVG80_01655 | KH domain-containing protein | 0.67 | 3.41E-02 | 1.32 | 3.32E-05 | 0.76 | 2.07E-02 | 0.78 | 2.90E-02 |
| DVG80_00884 | 50S ribosomal protein L20 | 0.75 | 1.72E-02 | 1.09 | 6.72E-04 | 0.77 | 1.81E-02 | 0.77 | 2.59E-02 |
| DVG80_01656 | 30S ribosomal protein S16 | 0.70 | 2.22E-02 | 0.94 | 2.63E-03 | 0.75 | 1.74E-02 | 0.72 | 3.17E-02 |
| DVG80_01625 | 30S ribosomal protein S2 | 0.82 | 1.07E-03 | 1.07 | 1.59E-05 | 0.81 | 1.48E-03 | 0.71 | 1.02E-02 |
| DVG80_03773 | ABC transporter substrate-binding protein | 0.92 | 3.72E-04 | 1.12 | 1.05E-05 | 1.31 | 1.10E-07 | 0.68 | 1.89E-02 |
| DVG80_01199 | DNA-directed RNA polymerase subunit omega | 0.66 | 2.26E-02 | 0.69 | 2.45E-02 | 0.67 | 2.88E-02 | 0.66 | 4.46E-02 |
| DVG80_00329 | 50S ribosomal protein L27 | 0.83 | 3.19E-03 | 0.93 | 1.10E-03 | 0.73 | 1.18E-02 | 0.66 | 3.65E-02 |
| DVG80_02488 | 50S ribosomal protein L11 | 0.65 | 1.54E-02 | 0.74 | 7.74E-03 | 0.66 | 1.85E-02 | 0.60 | 4.63E-02 |
| DVG80_03122 | acyl-CoA dehydrogenase | -1.47 | 5.27E-06 | -1.15 | 2.17E-04 | -1.03 | 8.58E-04 | -0.73 | 4.00E-02 |
| DVG80_02617 | hypothetical protein | -0.60 | 4.72E-02 | -0.77 | 1.60E-02 | -0.70 | 2.94E-02 | -0.74 | 4.75E-02 |
| DVG80_01379 | UDP-glucose 4-epimerase GalE | -0.89 | 9.69E-04 | -0.84 | 2.46E-03 | -0.61 | 3.47E-02 | -0.75 | 1.69E-02 |
| DVG80_02219 | GMP synthase (glutamine-hydrolyzing) | -0.73 | 1.03E-03 | -0.73 | 1.68E-03 | -0.80 | 4.23E-04 | -0.76 | 2.13E-03 |
| DVG80_00206 | hypothetical protein | -0.61 | 3.22E-02 | -0.88 | 2.86E-03 | -1.17 | 3.88E-05 | -0.77 | 2.88E-02 |
| DVG80_00984 | malate synthase G | -1.33 | 2.70E-05 | -0.84 | 7.69E-03 | -0.75 | 1.73E-02 | -0.79 | 2.05E-02 |
| DVG80_05339 | LysR family transcriptional regulator | -0.92 | 3.50E-03 | -0.78 | 1.61E-02 | -0.83 | 8.81E-03 | -0.82 | 4.71E-02 |
| DVG80_02590 | histidine kinase | -0.78 | 1.31E-02 | -1.00 | 1.90E-03 | -1.07 | 7.66E-04 | -0.82 | 4.28E-02 |
| DVG80_01252 | CBS domain-containing protein | -0.95 | 6.52E-03 | -0.81 | 2.12E-02 | -0.85 | 1.47E-02 | -0.84 | 4.75E-02 |
| DVG80_04084 | peptide-methionine (S)-S-oxide reductase | -0.63 | 4.29E-02 | -0.74 | 2.51E-02 | -0.81 | 1.18E-02 | -0.85 | 1.89E-02 |
| DVG80_03745 | non-ribosomal peptide synthetase | -0.81 | 2.04E-02 | -0.97 | 6.62E-03 | -0.97 | 5.05E-03 | -0.86 | 2.73E-02 |
| Gene | Description | 5 minutes | | 30 minutes | | 60 minutes | | 120 minutes | |
|  |  | log_2_FC | p-value (BH) | log_2_FC | p-value (BH) | log_2_FC | p-value (BH) | log_2_FC | p-value (BH) |
| DVG80_04385 | DNA replication and repair protein RecF | -0.65 | 2.28E-02 | -0.75 | 1.17E-02 | -0.65 | 3.34E-02 | -0.86 | 1.57E-02 |
| DVG80_05858 | MFS transporter | -0.73 | 2.05E-02 | -0.81 | 1.30E-02 | -0.68 | 3.92E-02 | -0.87 | 1.63E-02 |
| DVG80_03805 | anion transporter | -0.62 | 3.79E-02 | -0.70 | 2.63E-02 | -0.63 | 4.82E-02 | -0.89 | 1.72E-02 |
| DVG80_01492 | lipoate--protein ligase | -0.81 | 9.42E-03 | -0.96 | 2.56E-03 | -1.00 | 1.41E-03 | -0.91 | 1.70E-02 |
| DVG80_05167 | alpha/beta hydrolase | -1.15 | 6.66E-04 | -1.09 | 9.79E-04 | -1.03 | 1.64E-03 | -0.91 | 4.21E-02 |
| DVG80_01764 | GTP 3'%2C8-cyclase MoaA | -0.68 | 1.08E-02 | -1.04 | 1.17E-04 | -0.84 | 1.95E-03 | -0.92 | 3.99E-03 |
| DVG80_06279 | fumarate hydratase | -0.80 | 4.10E-03 | -0.79 | 6.69E-03 | -0.78 | 6.44E-03 | -0.92 | 3.30E-03 |
| DVG80_00786 | GNAT family N-acetyltransferase | -1.27 | 6.34E-04 | -1.51 | 3.04E-05 | -0.97 | 5.90E-03 | -0.94 | 3.08E-02 |
| DVG80_05863 | iron-siderophore ABC transporter substrate-binding protein | -0.64 | 4.90E-02 | -0.74 | 3.18E-02 | -0.85 | 1.27E-02 | -0.96 | 3.31E-02 |
| DVG80_02096 | N-acyl-L-amino acid amidohydrolase | -0.75 | 3.23E-03 | -1.04 | 5.81E-05 | -1.00 | 8.38E-05 | -0.96 | 1.59E-03 |
| DVG80_05577 | acyl-CoA dehydrogenase | -1.59 | 3.53E-05 | -1.18 | 1.20E-03 | -2.03 | 4.21E-07 | -0.97 | 4.37E-02 |
| DVG80_03104 | threonylcarbamoyl-AMP synthase | -0.90 | 5.75E-03 | -1.07 | 1.07E-03 | -0.74 | 2.66E-02 | -0.97 | 1.84E-02 |
| DVG80_05061 | ubiquinone biosynthesis protein | -0.95 | 6.41E-03 | -1.43 | 5.24E-05 | -1.08 | 1.75E-03 | -0.99 | 3.00E-02 |
| DVG80_05728 | MerR family DNA-binding transcriptional regulator | -0.74 | 1.42E-02 | -1.05 | 6.19E-04 | -0.82 | 8.07E-03 | -1.00 | 8.30E-03 |
| DVG80_04915 | TetR family transcriptional regulator | -0.86 | 1.74E-02 | -0.86 | 1.88E-02 | -0.72 | 4.85E-02 | -1.01 | 2.71E-02 |
| DVG80_05939 | transcriptional regulator | -0.98 | 1.63E-02 | -1.36 | 8.67E-04 | -1.13 | 4.09E-03 | -1.02 | 2.37E-02 |
| DVG80_04619 | TetR family transcriptional regulator | -1.06 | 6.33E-03 | -1.04 | 6.56E-03 | -1.09 | 3.99E-03 | -1.02 | 4.82E-02 |
| DVG80_02165 | LysR family transcriptional regulator | -0.67 | 3.18E-02 | -0.78 | 1.85E-02 | -0.92 | 4.59E-03 | -1.02 | 2.84E-02 |
| DVG80_00304 | hypothetical protein | -1.01 | 8.68E-04 | -0.90 | 3.31E-03 | -0.94 | 1.74E-03 | -1.03 | 3.30E-03 |
| DVG80_04880 | putative benzaldehyde dehydrogenase | -1.36 | 1.10E-03 | -1.39 | 5.51E-04 | -1.39 | 5.58E-04 | -1.03 | 4.18E-02 |
| DVG80_04735 | hypothetical protein | -0.72 | 3.17E-02 | -0.82 | 2.07E-02 | -0.70 | 4.82E-02 | -1.04 | 2.49E-02 |
| DVG80_03374 | M56 family peptidase | -0.80 | 1.89E-02 | -0.74 | 3.63E-02 | -1.16 | 8.74E-04 | -1.06 | 2.54E-02 |
| DVG80_04422 | hypothetical protein | -1.16 | 1.37E-04 | -1.02 | 6.78E-04 | -0.92 | 1.91E-03 | -1.06 | 2.41E-03 |
| DVG80_03863 | glutamine amidotransferase | -0.66 | 2.56E-02 | -1.05 | 6.16E-04 | -0.94 | 1.95E-03 | -1.06 | 7.30E-03 |
| Gene | Description | 5 minutes | | 30 minutes | | 60 minutes | | 120 minutes | |
|  |  | log_2_FC | p-value (BH) | log_2_FC | p-value (BH) | log_2_FC | p-value (BH) | log_2_FC | p-value (BH) |
| DVG80_01334 | hypothetical protein | -0.93 | 3.03E-03 | -0.90 | 4.69E-03 | -1.29 | 3.23E-05 | -1.06 | 7.65E-03 |
| DVG80_03228 | aspartate aminotransferase | -1.04 | 6.34E-04 | -1.17 | 8.96E-05 | -1.21 | 4.42E-05 | -1.07 | 2.55E-03 |
| DVG80_01565 | malate:quinone oxidoreductase | -1.18 | 9.04E-05 | -1.18 | 5.06E-05 | -0.85 | 4.00E-03 | -1.07 | 6.87E-04 |
| DVG80_03709 | hypothetical protein | -1.46 | 1.51E-08 | -1.70 | 7.95E-12 | -1.53 | 7.70E-10 | -1.08 | 2.22E-04 |
| DVG80_04417 | hypothetical protein | -1.08 | 5.72E-04 | -0.96 | 1.90E-03 | -0.88 | 4.32E-03 | -1.10 | 3.10E-03 |
| DVG80_06021 | histidine kinase | -1.04 | 3.52E-03 | -1.24 | 4.19E-04 | -1.08 | 1.72E-03 | -1.10 | 8.53E-03 |
| DVG80_04751 | MBL fold metallo-hydrolase | -1.28 | 4.23E-04 | -1.37 | 7.94E-05 | -1.08 | 1.51E-03 | -1.10 | 3.17E-03 |
| DVG80_02763 | O-succinylhomoserine sulfhydrylase | -0.69 | 1.62E-03 | -0.96 | 1.61E-05 | -0.96 | 9.98E-06 | -1.10 | 6.71E-06 |
| DVG80_02821 | oxidoreductase | -1.58 | 3.32E-05 | -1.36 | 1.82E-04 | -1.02 | 3.99E-03 | -1.11 | 9.04E-03 |
| DVG80_04178 | RNA-binding protein | -1.09 | 6.22E-03 | -0.93 | 1.72E-02 | -0.81 | 3.26E-02 | -1.13 | 3.60E-02 |
| DVG80_05951 | phospholipase | -1.24 | 1.03E-03 | -1.29 | 4.42E-04 | -1.15 | 1.42E-03 | -1.13 | 1.53E-02 |
| DVG80_00648 | quinolinate synthetase | -1.76 | 3.76E-05 | -1.71 | 3.35E-05 | -1.28 | 1.27E-03 | -1.14 | 9.63E-03 |
| DVG80_06234 | membrane protein | -0.77 | 4.92E-02 | -1.45 | 5.83E-04 | -1.21 | 2.90E-03 | -1.14 | 3.29E-02 |
| DVG80_05245 | allophanate hydrolase | -1.49 | 2.73E-03 | -1.39 | 3.22E-03 | -0.98 | 2.28E-02 | -1.14 | 4.63E-02 |
| DVG80_05128 | rubrerythrin family protein | -1.35 | 2.92E-03 | -2.13 | 1.83E-05 | -1.65 | 4.32E-04 | -1.15 | 4.65E-02 |
| DVG80_02264 | acyl-CoA dehydrogenase | -2.03 | 3.38E-05 | -2.42 | 2.27E-06 | -0.80 | 4.91E-02 | -1.16 | 2.13E-02 |
| DVG80_05905 | aldo/keto reductase | -1.07 | 1.63E-02 | -0.94 | 2.77E-02 | -0.85 | 3.91E-02 | -1.16 | 2.36E-02 |
| DVG80_04936 | succinate dehydrogenase flavoprotein subunit | -0.90 | 4.54E-02 | -1.13 | 1.48E-02 | -0.84 | 4.74E-02 | -1.18 | 4.40E-02 |
| DVG80_06018 | LLM class F420-dependent oxidoreductase | -1.26 | 1.48E-04 | -1.37 | 1.81E-05 | -1.15 | 2.59E-04 | -1.18 | 1.98E-03 |
| DVG80_01253 | cell filamentation protein | -1.31 | 7.23E-04 | -1.40 | 2.15E-04 | -1.23 | 9.91E-04 | -1.18 | 2.43E-02 |
| DVG80_06752 | molybdopterin biosynthesis protein MoeY | -0.73 | 4.13E-02 | -0.94 | 1.19E-02 | -1.12 | 2.49E-03 | -1.18 | 1.86E-02 |
| DVG80_02064 | hypothetical protein | -0.95 | 1.47E-02 | -1.60 | 5.91E-05 | -1.07 | 4.59E-03 | -1.20 | 1.29E-02 |
| DVG80_05246 | allophanate hydrolase | -1.58 | 2.30E-03 | -1.78 | 7.90E-04 | -4.24 | 3.05E-06 | -1.22 | 4.21E-02 |
| DVG80_01417 | membrane protein | -0.70 | 8.69E-03 | -0.97 | 2.91E-04 | -1.29 | 6.34E-07 | -1.23 | 1.08E-04 |
| DVG80_00411 | glycine--tRNA ligase | -0.91 | 1.48E-04 | -0.91 | 1.71E-04 | -0.94 | 8.20E-05 | -1.24 | 1.65E-06 |
| Gene | Description | 5 minutes | | 30 minutes | | 60 minutes | | 120 minutes | |
|  |  | log_2_FC | p-value (BH) | log_2_FC | p-value (BH) | log_2_FC | p-value (BH) | log_2_FC | p-value (BH) |
| DVG80_06612 | hypothetical protein | -0.73 | 1.63E-02 | -0.88 | 4.94E-03 | -0.85 | 5.49E-03 | -1.24 | 7.28E-04 |
| DVG80_05647 | amidophosphoribosyltransferase | -0.97 | 1.46E-05 | -1.04 | 2.55E-06 | -1.13 | 1.76E-07 | -1.25 | 7.61E-08 |
| DVG80_05213 | ATP-dependent protease | -1.36 | 8.72E-05 | -1.32 | 9.09E-05 | -1.12 | 8.36E-04 | -1.29 | 7.65E-03 |
| DVG80_02633 | isocitrate lyase | -1.39 | 6.30E-06 | -1.68 | 1.03E-08 | -1.05 | 3.54E-04 | -1.31 | 3.21E-05 |
| DVG80_05677 | urease subunit beta | -1.07 | 1.01E-02 | -2.10 | 2.09E-06 | -1.41 | 6.58E-04 | -1.31 | 7.88E-03 |
| DVG80_05786 | CdaR family transcriptional regulator | -1.28 | 7.61E-04 | -1.78 | 2.30E-06 | -1.95 | 5.10E-07 | -1.32 | 6.40E-03 |
| DVG80_03399 | hydrolase | -1.27 | 1.62E-03 | -2.41 | 8.00E-09 | -1.54 | 9.02E-05 | -1.32 | 4.68E-03 |
| DVG80_04747 | SARP family transcriptional regulator | -1.44 | 3.08E-04 | -1.30 | 7.13E-04 | -0.83 | 2.49E-02 | -1.34 | 1.69E-02 |
| DVG80_02086 | glycerol-3-phosphate dehydrogenase/oxidase | -1.44 | 7.69E-06 | -1.40 | 6.37E-06 | -1.22 | 6.96E-05 | -1.34 | 5.48E-04 |
| DVG80_05785 | 1-pyrroline-5-carboxylate dehydrogenase | -1.26 | 1.27E-02 | -1.57 | 2.85E-03 | -1.14 | 1.48E-02 | -1.35 | 2.48E-02 |
| DVG80_01081 | hypothetical protein | -1.14 | 2.17E-03 | -1.43 | 8.36E-05 | -1.24 | 5.05E-04 | -1.36 | 1.71E-03 |
| DVG80_01366 | cation:proton antiporter | -0.90 | 3.52E-03 | -1.11 | 3.47E-04 | -1.26 | 3.89E-05 | -1.37 | 9.87E-04 |
| DVG80_05060 | CdaR family transcriptional regulator | -1.46 | 2.43E-03 | -1.72 | 4.19E-04 | -1.18 | 7.26E-03 | -1.37 | 2.78E-02 |
| DVG80_01034 | Pup--protein ligase 2 | -1.39 | 4.04E-06 | -1.32 | 5.86E-06 | -1.12 | 9.38E-05 | -1.40 | 2.73E-05 |
| DVG80_01778 | phosphoglucomutase%2C alpha-D-glucose phosphate-specific | -1.28 | 5.07E-03 | -1.37 | 1.90E-03 | -1.15 | 6.15E-03 | -1.41 | 6.12E-03 |
| DVG80_00140 | hypothetical protein | -1.47 | 3.94E-04 | -1.28 | 1.09E-03 | -0.98 | 9.41E-03 | -1.42 | 4.38E-03 |
| DVG80_05952 | pyridine nucleotide-disulfide oxidoreductase | -1.46 | 2.22E-05 | -1.39 | 2.87E-05 | -1.11 | 7.46E-04 | -1.42 | 2.04E-03 |
| DVG80_05247 | MarR family transcriptional regulator | -0.87 | 4.24E-02 | -2.18 | 2.18E-05 | -1.30 | 3.58E-03 | -1.43 | 1.53E-02 |
| DVG80_01850 | FadR family transcriptional regulator | -1.51 | 2.24E-04 | -1.60 | 5.01E-05 | -1.34 | 5.21E-04 | -1.44 | 5.71E-03 |
| DVG80_01367 | potassium transporter TrkA | -1.21 | 3.94E-05 | -1.33 | 2.98E-06 | -1.23 | 1.30E-05 | -1.49 | 3.34E-05 |
| DVG80_05714 | cell division protein FtsW | -0.91 | 1.10E-03 | -1.12 | 5.66E-05 | -1.01 | 2.60E-04 | -1.49 | 6.41E-06 |
| DVG80_02809 | hypothetical protein | -0.93 | 1.63E-02 | -0.96 | 1.30E-02 | -0.85 | 2.43E-02 | -1.50 | 3.13E-03 |
| DVG80_06758 | transcriptional regulator | -0.70 | 4.72E-02 | -1.30 | 5.27E-04 | -0.86 | 1.80E-02 | -1.55 | 4.14E-03 |
| DVG80_00074 | methylmalonate-semialdehyde dehydrogenase (CoA acylating) | -1.73 | 2.38E-07 | -1.79 | 3.26E-08 | -1.06 | 1.04E-03 | -1.56 | 7.78E-06 |
| Gene | Description | 5 minutes | | 30 minutes | | 60 minutes | | 120 minutes | |
|  |  | log_2_FC | p-value (BH) | log_2_FC | p-value (BH) | log_2_FC | p-value (BH) | log_2_FC | p-value (BH) |
| DVG80_00141 | ParA family protein | -1.20 | 1.45E-03 | -1.21 | 8.87E-04 | -0.76 | 3.44E-02 | -1.58 | 3.41E-04 |
| DVG80_04739 | diacylglycerol kinase | -1.51 | 4.90E-04 | -1.38 | 8.01E-04 | -1.37 | 8.43E-04 | -1.59 | 7.76E-03 |
| DVG80_00267 | copper resistance protein CopD | -1.18 | 1.37E-04 | -1.73 | 4.66E-09 | -1.94 | 9.27E-11 | -1.59 | 2.00E-06 |
| DVG80_04459 | haloacid dehalogenase%2C type II | -1.33 | 2.30E-03 | -1.12 | 6.69E-03 | -0.85 | 3.31E-02 | -1.63 | 1.02E-02 |
| DVG80_00205 | hypothetical protein | -0.97 | 1.38E-03 | -1.51 | 3.44E-07 | -1.52 | 2.60E-07 | -1.67 | 6.52E-06 |
| DVG80_03678 | flotillin | -0.93 | 2.89E-02 | -1.88 | 6.72E-05 | -1.22 | 4.45E-03 | -1.69 | 3.33E-03 |
| DVG80_05676 | urease subunit gamma | -1.16 | 2.40E-03 | -2.00 | 1.98E-07 | -1.62 | 1.77E-05 | -1.75 | 1.95E-04 |
| DVG80_02688 | amino acid dehydrogenase | -1.19 | 4.88E-03 | -1.40 | 6.88E-04 | -1.33 | 1.04E-03 | -1.75 | 2.41E-04 |
| DVG80_04167 | histidine-type phosphatase | -2.39 | 3.97E-05 | -3.36 | 2.06E-06 | -2.19 | 2.22E-04 | -1.80 | 7.63E-03 |
| DVG80_05864 | hypothetical protein | -1.01 | 4.23E-03 | -1.29 | 2.09E-04 | -1.29 | 1.96E-04 | -1.86 | 3.18E-05 |
| DVG80_04882 | FAD-dependent oxidoreductase | -0.76 | 2.94E-02 | -2.49 | 1.14E-11 | -1.23 | 4.27E-04 | -1.90 | 8.26E-07 |
| DVG80_01355 | DNA-binding protein | -1.95 | 5.30E-08 | -2.10 | 3.61E-09 | -2.06 | 1.27E-08 | -1.94 | 1.14E-05 |
| DVG80_05737 | LuxR family transcriptional regulator | -0.92 | 5.63E-03 | -1.24 | 1.85E-04 | -1.60 | 1.84E-06 | -1.97 | 6.74E-05 |
| DVG80_02263 | TetR family transcriptional regulator | -2.25 | 6.96E-08 | -2.72 | 4.36E-10 | -2.22 | 2.38E-07 | -2.02 | 4.43E-05 |
| DVG80_05784 | proline dehydrogenase | -1.43 | 9.75E-04 | -1.86 | 2.41E-05 | -1.74 | 8.66E-05 | -2.03 | 8.51E-03 |
| DVG80_02632 | 3-hydroxybutyryl-CoA dehydrogenase | -2.27 | 3.40E-09 | -2.10 | 3.94E-08 | -2.14 | 4.98E-08 | -2.06 | 4.81E-05 |
| DVG80_00189 | hypothetical protein | -1.02 | 4.39E-04 | -1.34 | 1.91E-06 | -1.81 | 1.04E-10 | -2.10 | 6.11E-11 |
| DVG80_05614 | hypothetical protein | -1.90 | 2.97E-08 | -2.13 | 4.39E-10 | -2.23 | 1.72E-10 | -2.12 | 1.66E-06 |
| DVG80_01099 | transcriptional regulator | -0.78 | 1.27E-02 | -1.07 | 6.76E-04 | -1.38 | 7.04E-06 | -2.14 | 7.18E-09 |
| DVG80_06751 | molybdopterin biosynthesis protein MoeY | -0.91 | 6.36E-03 | -1.20 | 3.12E-04 | -1.32 | 6.14E-05 | -2.18 | 1.81E-06 |
| DVG80_01963 | molybdopterin biosynthesis protein MoeY | -0.74 | 3.85E-02 | -1.40 | 1.43E-04 | -1.22 | 7.71E-04 | -2.22 | 3.11E-06 |
| DVG80_03007 | hypothetical protein | -0.95 | 3.34E-02 | -1.86 | 2.56E-04 | -2.58 | 1.30E-05 | -2.27 | 9.87E-04 |
| DVG80_04738 | hypothetical protein | -2.86 | 3.64E-10 | -4.44 | 9.41E-14 | -3.25 | 3.38E-10 | -2.44 | 2.03E-03 |
| DVG80_03010 | histidine kinase | -2.49 | 3.27E-14 | -2.40 | 4.34E-13 | -2.55 | 9.86E-14 | -2.58 | 5.47E-08 |
| Gene | Description | 5 minutes | | 30 minutes | | 60 minutes | | 120 minutes | |
|  |  | log_2_FC | p-value (BH) | log_2_FC | p-value (BH) | log_2_FC | p-value (BH) | log_2_FC | p-value (BH) |
| DVG80_04442 | pyruvate dehydrogenase (acetyl-transferring) E1 component subunit alpha | -0.80 | 2.39E-02 | -2.15 | 5.30E-09 | -1.88 | 3.45E-07 | -2.71 | 8.86E-09 |
| DVG80_06392 | GntR family transcriptional regulator | -1.36 | 8.85E-03 | -3.84 | 2.82E-06 | -3.93 | 2.10E-06 | -2.72 | 2.93E-04 |
| DVG80_00647 | NUDIX hydrolase | -1.81 | 3.09E-05 | -2.88 | 5.51E-10 | -1.53 | 2.23E-04 | -2.72 | 1.63E-06 |
| DVG80_06017 | universal stress protein | -2.69 | 7.05E-09 | -3.10 | 2.32E-10 | -3.15 | 1.88E-10 | -2.86 | 6.01E-08 |
| DVG80_04076 | bacterioferritin | -0.88 | 2.86E-02 | -3.34 | 2.54E-12 | -4.00 | 1.84E-16 | -3.01 | 9.40E-11 |
| DVG80_05717 | VIT family protein | -2.55 | 1.42E-11 | -3.97 | 1.40E-21 | -3.98 | 8.90E-22 | -4.18 | 1.81E-13 |
| DVG80_04092 | hypothetical protein | -4.09 | 1.25E-13 | -5.49 | 5.98E-22 | -4.82 | 1.13E-17 | -4.35 | 3.57E-11 |
| DVG80_05658 | cytochrome ubiquinol oxidase subunit I | -1.21 | 1.21E-02 | -4.45 | 2.78E-11 | -4.63 | 3.99E-12 | -4.77 | 9.10E-12 |

**Table S5.** Selected host genes involved in the TCA and glycoxylate cycles. Log2FC and p-values are shown for all time points as compared to baseline (0 minutes). Entries indicated in bold are those that are involved in both pathways. Malate synthase has been italicized to indicate that it is unique to the glycoxylate cycle. Shaded cells indicate DE.

| Gene | Description | T5  Log_2_FC p-value | | T30  Log_2_FC p-value | | T60  Log_2_FC p-value | | T120  Log_2_FC p-value | |
| --- | --- | --- | --- | --- | --- | --- | --- | --- | --- |
| DVG80_00049 | pyruvate dehydrogenase (acetyl-transferring) E1 component subunit alpha | -0.19 | 0.6269 | -0.90 | 0.0457 | -0.11 | 0.8243 | -0.24 | 0.6623 |
| DVG80_01565 | malate:quinone oxidoreductase | -1.18 | 9.04E-05 | -1.18 | 5.06E-05 | -0.85 | 0.0040 | -1.07 | 6.87E-04 |
| DVG80_03165 | phosphoenolpyruvate carboxykinase | -0.43 | 0.1162 | -0.88 | 0.0012 | -0.59 | 0.0416 | -0.88 | 0.0024 |
| DVG80_04442 | pyruvate dehydrogenase (acetyl-transferring) E1 component subunit alpha | -0.80 | 0.0239 | -2.15 | 5.30E-09 | -1.88 | 3.45E-07 | -2.71 | 8.86E-09 |
| DVG80_04936 | succinate dehydrogenase flavoprotein subunit | -0.90 | 0.0454 | -1.13 | 0.0148 | -0.84 | 0.0474 | -1.18 | 0.0440 |
| DVG80_02110 | succinate dehydrogenase%2C cytochrome b556 subunit | -0.13 | 0.7187 | -0.56 | 0.0747 | -0.31 | 0.3483 | -0.71 | 0.0290 |
| DVG80_02111 | succinate dehydrogenase | -0.10 | 0.8059 | -0.49 | 0.1605 | -0.15 | 0.6994 | -0.42 | 0.3104 |
| DVG80_02112 | succinate dehydrogenase flavoprotein subunit | -0.01 | 0.9863 | -0.42 | 0.1814 | -0.13 | 0.7157 | -0.42 | 0.2255 |
| DVG80_02113 | succinate dehydrogenase iron-sulfur subunit | -0.03 | 0.9470 | -0.50 | 0.1200 | -0.31 | 0.3490 | -0.39 | 0.2891 |
| DVG80_06254 | alpha-ketoglutarate decarboxylase | 0.36 | 0.3174 | -0.11 | 0.8088 | -0.19 | 0.6496 | -0.27 | 0.5975 |
| DVG80_06057 | succinyl-CoA ligase subunit beta | -0.13 | 0.6679 | -0.19 | 0.4709 | -0.01 | 0.9591 | -0.24 | 0.4127 |
| DVG80_06058 | succinate--CoA ligase subunit alpha | -0.06 | 0.8604 | -0.16 | 0.5551 | -0.06 | 0.8347 | -0.29 | 0.3207 |
| DVG80_06279 | **fumarate hydratase** | **-0.80** | **0.0041** | **-0.79** | **0.0067** | **-0.78** | **0.0064** | **-0.92** | **0.0033** |
| DVG80_01274 | **malate dehydrogenase** | **-0.48** | **0.1530** | **-0.86** | **0.0174** | **-0.86** | **0.0147** | **-0.64** | **0.1137** |
| DVG80_02633 | **isocitrate lyase** | **-1.39** | **6.30E-06** | **-1.68** | **1.03E-08** | **-1.05** | **3.54E-04** | **-1.31** | **3.21E-05** |
| DVG80_01136 | **aconitate hydratase** | 0.01 | 0.9822 | 0.06 | 0.8687 | 0.18 | 0.5831 | 0.04 | 0.9354 |
| DVG80_05876 | **citrate synthase** | -0.25 | 0.4625 | -0.30 | 0.3994 | -0.29 | 0.4130 | -0.53 | 0.1618 |
| *DVG80_00984* | *malate synthase* | *-1.33* | *2.70E-05* | *-0.84* | *0.0077* | *-0.75* | *0.0173* | *-0.79* | *0.0205* |

**Table S6.** Selected host genes related to metal homeostasis. All time points are with respect to the baseline. Blue highlights indicated significantly up-regulated DE genes, while grey highlights indicate those that were significantly down-regulated.

| Gene | Annotations | T5vsT0 | T30vsT0 | T60vsT0 | T120vsT0 |
| --- | --- | --- | --- | --- | --- |
| DVG80_04450 | metal ABC transporter substrate-binding protein; mntC | 2.04 (0.00031) | 2.18 (0.00016) | 1.75 (0.00119) | 2.8 (1.92e-05) |
| DVG80_06203 | iron transporter | 1.97 (1.66e-06) | 2.68 (1.64e-10) | 2.99 (4.04e-12) | 2.68 (5.47e-08) |
| DVG80_05365 | divalent metal cation transporter; mntH | 1.65 (0.009) | 1.37 (0.004) | 1.53 (0.00193) | 1.53 (0.01) |
| DVG80_03824 | ABC transporter substrate-binding protein | 0.8 (0.04) | 1.37 (0.0007) | 1.14 (0.00367) | 1.46 (0.00139) |
| DVG80_04452 | metal ABC transporter permease; mntB | 0.88 (0.04) | 1.08 (0.02) | 0.89 (0.03) | 1.21 (0.02) |
| DVG80_04451 | ABC transporter; mntA | 0.87 (0.05) | 1.12 (0.02) | 0.86 (0.05) | 1.17 (0.03) |
| DVG80_01342 | iron-siderophore ABC transporter substrate-binding protein | 0.54 (0.14) | 1.7 (3.53e-05) | 2.28 (1.56e-07) | 2.06 (3.03e-05) |
| DVG80_01095 | iron-siderophore ABC transporter permease protein | 0.34 (0.29) | 1.62 (9.1e-08) | 1.8 (3.4e-09) | 1.6 (2.33e-05) |
| DVG80_00319 | iron-siderophore ABC transporter substrate-binding protein | -0.03 (0.96) | 0.95 (0.0038) | 1.16 (0.00026) | 1.43 (0.00027) |
| DVG80_03209 | iron ABC transporter substrate-binding protein | 0.28 (0.47) | 0.93 (0.03) | 1.24 (0.0041) | 0.44 (0.41) |
| DVG80_00998 | iron ABC transporter permease | -0.01 (0.99) | 0.57 (0.1) | 0.61 (0.07) | 0.84 (0.04) |
| DVG80_01747 | iron siderophore-binding protein | 0.31 (0.42) | 0.43 (0.33) | 0.96 (0.03) | 0.05 (0.93) |
| DVG80_04674 | iron ABC transporter substrate-binding protein | -0.2 (0.62) | -0.97 (0.03) | -0.15 (0.74) | -0.63 (0.21) |
| DVG80_05863 | iron-siderophore ABC transporter substrate-binding protein | -0.64 (0.05) | -0.74 (0.03) | -0.85 (0.01) | -0.96 (0.03) |
| DVG80_05659 | cytochrome d ubiquinol oxidase subunit II | -0.15 (0.72) | -2.72 (4.5e-06) | -2.98 (1.74e-06) | -2.11 (0.00049) |
| DVG80_05658 | cytochrome ubiquinol oxidase subunit I | -1.21 (0.01) | -4.45 (2.78e-11) | -4.63 (3.99e-12) | -4.77 (9.1e-12) |
| DVG80_04076 | bacterioferritin | -0.88 (0.03) | -3.34 (2.54e-12) | -4.0 (1.84e-16) | -3.01 (9.4e-11) |
| DVG80_04068 | bacterioferritin | -0.20 (0.55) | -0.10 (0.81) | -0.10 (0.79) | -0.33 (0.45) |
| DVG80_05717 | VIT family protein | -2.55 (1.42e-11) | -3.97 (1.40e-21) | -3.98 (8.9e-22) | -4.18 (1.81e-13) |
| DVG80_04092 | UPF0016 domain-containing protein | -4.09 (1.25e-13) | -5.49 (5.98e-22) | -4.82 (1.13e-17) | -4.35 (3.57e-11) |
| DVG80_03017 | transcriptional repressor; furA | -0.44 (NA) | -0.46 (0.3) | -0.07 (0.89) | 0.05 (0.94) |
| DVG80_00409 | transcriptional repressor; furB | -0.29 (0.39) | -0.23 (0.54) | -0.12 (0.77) | -0.63 (0.17) |
| DVG80_01378 | dihydrofolate reductase (Genbank); ideR (eggNOG; InterProScan) | -0.31 (0.30) | -0.34 (0.30) | -0.39 (0.23) | -0.30 (0.43) |

**Table S7.** Enriched GO terms from the Biological Process ontology for each time point as compared to the baseline. All enriched terms were up-regulated as compared to baseline. P-values were adjusted using the Benjamini-Hochberg method. The Summary Term is the grouping used in Figure 6.

| GO ID | Description | Adjusted p-value | Gene  Count | Time | Ontology | Summary Term |
| --- | --- | --- | --- | --- | --- | --- |
| GO:0000018 | regulation of DNA recombination | 3.44E-02 | 2 | T5_T0 | BP | negative regulation of strand invasion |
| GO:0006139 | nucleobase-containing compound metabolic process | 1.72E-02 | 38 | T5_T0 | BP | nucleic acid phosphodiester bond hydrolysis |
| GO:0006259 | DNA metabolic process | 4.41E-08 | 22 | T5_T0 | BP | DNA repair |
| GO:0006260 | DNA replication | 5.08E-06 | 13 | T5_T0 | BP | DNA replication |
| GO:0006261 | DNA-templated DNA replication | 1.82E-04 | 7 | T5_T0 | BP | DNA replication |
| GO:0006281 | DNA repair | 3.90E-08 | 19 | T5_T0 | BP | DNA repair |
| GO:0006302 | double-strand break repair | 2.37E-02 | 4 | T5_T0 | BP | DNA repair |
| GO:0006310 | DNA recombination | 3.24E-03 | 9 | T5_T0 | BP | DNA repair |
| GO:0006396 | RNA processing | 3.40E-02 | 9 | T5_T0 | BP | RNA processing |
| GO:0006401 | RNA catabolic process | 3.35E-02 | 4 | T5_T0 | BP | histidine catabolic process |
| GO:0006412 | translation | 3.18E-06 | 22 | T5_T0 | BP | translation |
| GO:0006518 | peptide metabolic process | 1.25E-06 | 23 | T5_T0 | BP | translation |
| GO:0006548 | histidine catabolic process | 2.34E-02 | 3 | T5_T0 | BP | histidine catabolic process |
| GO:0006950 | response to stress | 9.59E-03 | 18 | T5_T0 | BP | cellular response to stress |
| GO:0006974 | cellular response to DNA damage stimulus | 1.13E-05 | 15 | T5_T0 | BP | DNA repair |
| GO:0006996 | organelle organization | 1.53E-02 | 7 | T5_T0 | BP | ribosome biogenesis |
| GO:0009059 | macromolecule biosynthetic process | 6.03E-06 | 39 | T5_T0 | BP | translation |
| GO:0009307 | DNA restriction-modification system | 2.94E-03 | 5 | T5_T0 | BP | DNA restriction-modification system |
| GO:0009314 | response to radiation | 3.44E-02 | 2 | T5_T0 | BP | UV protection |
| GO:0009411 | response to UV | 3.44E-02 | 2 | T5_T0 | BP | UV protection |
| GO:0009416 | response to light stimulus | 3.44E-02 | 2 | T5_T0 | BP | UV protection |
| GO:0009432 | SOS response | 7.22E-03 | 5 | T5_T0 | BP | SOS response |
| GO:0009650 | UV protection | 3.44E-02 | 2 | T5_T0 | BP | UV protection |
| GO:0009892 | negative regulation of metabolic process | 4.64E-02 | 7 | T5_T0 | BP | negative regulation of metabolic process |
| GO:0010467 | gene expression | 6.66E-06 | 36 | T5_T0 | BP | gene expression |
| GO:0015942 | formate metabolic process | 3.83E-03 | 3 | T5_T0 | BP | formate metabolic process |
| GO:0019538 | protein metabolic process | 9.83E-05 | 30 | T5_T0 | BP | translation |
| GO:0019556 | histidine catabolic process to glutamate and formamide | 1.17E-02 | 3 | T5_T0 | BP | formamide metabolic process |
| GO:0019557 | histidine catabolic process to glutamate and formate | 1.17E-02 | 3 | T5_T0 | BP | formate metabolic process |
| GO:0022613 | ribonucleoprotein complex biogenesis | 4.67E-03 | 9 | T5_T0 | BP | ribosome biogenesis |
| GO:0033554 | cellular response to stress | 6.55E-04 | 16 | T5_T0 | BP | cellular response to stress |
| GO:0040007 | growth | 6.34E-03 | 41 | T5_T0 | BP | growth |
| GO:0042254 | ribosome biogenesis | 4.67E-03 | 9 | T5_T0 | BP | ribosome biogenesis |
| GO:0043043 | peptide biosynthetic process | 3.65E-06 | 22 | T5_T0 | BP | translation |
| GO:0043170 | macromolecule metabolic process | 3.90E-08 | 122 | T5_T0 | BP | macromolecule metabolic process |
| GO:0043603 | cellular amide metabolic process | 3.80E-06 | 27 | T5_T0 | BP | amide biosynthetic process |
| GO:0043604 | amide biosynthetic process | 1.04E-04 | 22 | T5_T0 | BP | amide biosynthetic process |
| GO:0043606 | formamide metabolic process | 3.83E-03 | 3 | T5_T0 | BP | formamide metabolic process |
| GO:0044271 | cellular nitrogen compound biosynthetic process | 2.62E-02 | 36 | T5_T0 | BP | amide biosynthetic process |
| GO:0045910 | negative regulation of DNA recombination | 3.44E-02 | 2 | T5_T0 | BP | negative regulation of strand invasion |
| GO:0051103 | DNA ligation involved in DNA repair | 3.44E-02 | 2 | T5_T0 | BP | DNA ligation involved in DNA repair |
| GO:0051716 | cellular response to stimulus | 1.10E-02 | 17 | T5_T0 | BP | cellular response to stress |
| GO:0052805 | imidazole-containing compound catabolic process | 2.34E-02 | 3 | T5_T0 | BP | imidazole-containing compound catabolism |
| GO:0060542 | regulation of strand invasion | 3.44E-02 | 2 | T5_T0 | BP | negative regulation of strand invasion |
| GO:0060543 | negative regulation of strand invasion | 3.44E-02 | 2 | T5_T0 | BP | negative regulation of strand invasion |
| GO:0071826 | ribonucleoprotein complex subunit organization | 2.37E-02 | 4 | T5_T0 | BP | ribosome biogenesis |
| GO:0071897 | DNA biosynthetic process | 2.07E-04 | 6 | T5_T0 | BP | DNA repair |
| GO:0090304 | nucleic acid metabolic process | 4.33E-05 | 35 | T5_T0 | BP | macromolecule metabolic process |
| GO:0090305 | nucleic acid phosphodiester bond hydrolysis | 1.96E-04 | 12 | T5_T0 | BP | nucleic acid phosphodiester bond hydrolysis |
| GO:0000027 | ribosomal large subunit assembly | 1.39E-02 | 4 | T30_T0 | BP | ribosome biogenesis |
| GO:0006364 | rRNA processing | 1.33E-02 | 8 | T30_T0 | BP | rRNA processing |
| GO:0006396 | RNA processing | 1.71E-03 | 14 | T30_T0 | BP | RNA processing |
| GO:0006412 | translation | 2.45E-28 | 52 | T30_T0 | BP | translation |
| GO:0006413 | translational initiation | 4.17E-02 | 3 | T30_T0 | BP | translational initiation |
| GO:0006414 | translational elongation | 1.36E-03 | 4 | T30_T0 | BP | translation |
| GO:0006518 | peptide metabolic process | 1.55E-28 | 53 | T30_T0 | BP | translation |
| GO:0006996 | organelle organization | 3.45E-02 | 8 | T30_T0 | BP | ribosome biogenesis |
| GO:0009059 | macromolecule biosynthetic process | 1.37E-17 | 71 | T30_T0 | BP | translation |
| GO:0010467 | gene expression | 4.46E-25 | 77 | T30_T0 | BP | gene expression |
| GO:0016070 | RNA metabolic process | 1.38E-02 | 28 | T30_T0 | BP | RNA processing |
| GO:0016072 | rRNA metabolic process | 2.10E-02 | 8 | T30_T0 | BP | rRNA processing |
| GO:0019538 | protein metabolic process | 6.37E-18 | 61 | T30_T0 | BP | translation |
| GO:0022613 | ribonucleoprotein complex biogenesis | 4.78E-06 | 15 | T30_T0 | BP | ribosome biogenesis |
| GO:0022618 | ribonucleoprotein complex assembly | 7.38E-04 | 6 | T30_T0 | BP | ribosome biogenesis |
| GO:0034470 | ncRNA processing | 1.39E-02 | 12 | T30_T0 | BP | RNA processing |
| GO:0040007 | growth | 8.76E-05 | 61 | T30_T0 | BP | growth |
| GO:0042254 | ribosome biogenesis | 4.78E-06 | 15 | T30_T0 | BP | ribosome biogenesis |
| GO:0042255 | ribosome assembly | 7.38E-04 | 6 | T30_T0 | BP | ribosome assembly |
| GO:0042273 | ribosomal large subunit biogenesis | 7.38E-04 | 5 | T30_T0 | BP | ribosome biogenesis |
| GO:0043043 | peptide biosynthetic process | 4.24E-28 | 52 | T30_T0 | BP | translation |
| GO:0043170 | macromolecule metabolic process | 6.90E-14 | 174 | T30_T0 | BP | macromolecule metabolic process |
| GO:0043603 | cellular amide metabolic process | 3.79E-24 | 58 | T30_T0 | BP | amide biosynthetic process |
| GO:0043604 | amide biosynthetic process | 2.31E-24 | 54 | T30_T0 | BP | amide biosynthetic process |
| GO:0044085 | cellular component biogenesis | 8.35E-03 | 20 | T30_T0 | BP | ribosome biogenesis |
| GO:0044271 | cellular nitrogen compound biosynthetic process | 1.41E-09 | 70 | T30_T0 | BP | amide biosynthetic process |
| GO:0070925 | organelle assembly | 2.99E-03 | 6 | T30_T0 | BP | ribosome assembly |
| GO:0071826 | ribonucleoprotein complex subunit organization | 9.72E-05 | 7 | T30_T0 | BP | ribosome biogenesis |
| GO:0071840 | cellular component organization or biogenesis | 3.09E-02 | 23 | T30_T0 | BP | ribosome assembly |
| GO:0090304 | nucleic acid metabolic process | 1.85E-02 | 36 | T30_T0 | BP | macromolecule metabolic process |
| GO:1901566 | organonitrogen compound biosynthetic process | 1.08E-05 | 61 | T30_T0 | BP | translation |
| GO:0043043 | peptide biosynthetic process | 2.31E-13 | 33 | T60_T0 | BP | translation |
| GO:0006518 | peptide metabolic process | 3.96E-13 | 33 | T60_T0 | BP | translation |
| GO:0006412 | translation | 9.42E-13 | 32 | T60_T0 | BP | translation |
| GO:0043604 | amide biosynthetic process | 2.03E-11 | 34 | T60_T0 | BP | amide biosynthetic process |
| GO:0043603 | cellular amide metabolic process | 3.86E-10 | 35 | T60_T0 | BP | amide biosynthetic process |
| GO:0010467 | gene expression | 3.36E-09 | 45 | T60_T0 | BP | gene expression |
| GO:0019538 | protein metabolic process | 4.67E-06 | 35 | T60_T0 | BP | translation |
| GO:0009059 | macromolecule biosynthetic process | 7.85E-05 | 39 | T60_T0 | BP | translation |
| GO:0000027 | ribosomal large subunit assembly | 6.68E-03 | 4 | T60_T0 | BP | ribosome biogenesis |
| GO:0006996 | organelle organization | 4.23E-02 | 7 | T60_T0 | BP | ribosome biogenesis |
| GO:0022613 | ribonucleoprotein complex biogenesis | 6.15E-04 | 11 | T60_T0 | BP | ribosome biogenesis |
| GO:0022618 | ribonucleoprotein complex assembly | 3.85E-03 | 5 | T60_T0 | BP | ribosome biogenesis |
| GO:0040007 | growth | 3.53E-02 | 42 | T60_T0 | BP | growth |
| GO:0042254 | ribosome biogenesis | 6.15E-04 | 11 | T60_T0 | BP | ribosome biogenesis |
| GO:0042255 | ribosome assembly | 3.85E-03 | 5 | T60_T0 | BP | ribosome assembly |
| GO:0042273 | ribosomal large subunit biogenesis | 6.68E-03 | 4 | T60_T0 | BP | ribosome biogenesis |
| GO:0043170 | macromolecule metabolic process | 6.52E-03 | 95 | T60_T0 | BP | macromolecule metabolic process |
| GO:0043933 | protein-containing complex organization | 1.44E-02 | 8 | T60_T0 | BP | ribosome biogenesis |
| GO:0044271 | cellular nitrogen compound biosynthetic process | 1.49E-02 | 41 | T60_T0 | BP | amide biosynthetic process |
| GO:0065003 | protein-containing complex assembly | 3.53E-02 | 7 | T60_T0 | BP | ribosome biogenesis |
| GO:0070925 | organelle assembly | 1.06E-02 | 5 | T60_T0 | BP | ribosome assembly |
| GO:0071826 | ribonucleoprotein complex subunit organization | 5.05E-04 | 6 | T60_T0 | BP | ribosome biogenesis |
| GO:0006281 | DNA repair | 3.46E-02 | 16 | T120_T0 | BP | DNA repair |
| GO:0006412 | translation | 4.46E-12 | 42 | T120_T0 | BP | translation |
| GO:0006518 | peptide metabolic process | 3.20E-12 | 43 | T120_T0 | BP | translation |
| GO:0009059 | macromolecule biosynthetic process | 4.79E-04 | 58 | T120_T0 | BP | translation |
| GO:0010467 | gene expression | 9.32E-08 | 63 | T120_T0 | BP | gene expression |
| GO:0019538 | protein metabolic process | 6.88E-06 | 52 | T120_T0 | BP | translation |
| GO:0043043 | peptide biosynthetic process | 6.32E-12 | 42 | T120_T0 | BP | translation |
| GO:0043170 | macromolecule metabolic process | 3.26E-02 | 153 | T120_T0 | BP | macromolecule metabolic process |
| GO:0043603 | cellular amide metabolic process | 9.32E-08 | 45 | T120_T0 | BP | amide biosynthetic process |
| GO:0043604 | amide biosynthetic process | 1.40E-09 | 44 | T120_T0 | BP | amide biosynthetic process |
| GO:0071826 | ribonucleoprotein complex subunit organization | 1.83E-02 | 6 | T120_T0 | BP | ribosome biogenesis |

**Table S8.** Enriched GO terms from the Molecular Function ontology for each time point as compared to the baseline. All enriched terms were up-regulated as compared to baseline. P-values were adjusted using the Benjamini-Hochberg method. The Summary Term is the grouping used in Figure 6.

| GO ID | Description | Adjusted p-value | Gene  Count | Time | Ontology | Summary Term |
| --- | --- | --- | --- | --- | --- | --- |
| GO:0003676 | nucleic acid binding | 6.69E-18 | 67 | T5_T0 | MF | RNA binding |
| GO:0003678 | DNA helicase activity | 2.97E-02 | 5 | T5_T0 | MF | DNA-directed DNA polymerase activity |
| GO:0003684 | damaged DNA binding | 1.72E-02 | 4 | T5_T0 | MF | damaged DNA binding |
| GO:0003723 | RNA binding | 1.25E-06 | 25 | T5_T0 | MF | RNA binding |
| GO:0003724 | RNA helicase activity | 1.17E-02 | 4 | T5_T0 | MF | DNA-directed 5'-3' RNA polymerase activity |
| GO:0003735 | structural constituent of ribosome | 4.33E-08 | 19 | T5_T0 | MF | structural constituent of ribosome |
| GO:0003743 | translation initiation factor activity | 1.17E-02 | 3 | T5_T0 | MF | translation initiation factor activity |
| GO:0003887 | DNA-directed DNA polymerase activity | 4.04E-07 | 9 | T5_T0 | MF | DNA-directed DNA polymerase activity |
| GO:0004386 | helicase activity | 2.74E-04 | 10 | T5_T0 | MF | DNA-directed DNA polymerase activity |
| GO:0004518 | nuclease activity | 2.34E-04 | 12 | T5_T0 | MF | endonuclease activity |
| GO:0004519 | endonuclease activity | 7.91E-09 | 22 | T5_T0 | MF | endonuclease activity |
| GO:0004521 | endoribonuclease activity | 4.02E-02 | 4 | T5_T0 | MF | endonuclease activity |
| GO:0004527 | exonuclease activity | 1.62E-04 | 9 | T5_T0 | MF | endonuclease activity |
| GO:0005198 | structural molecule activity | 4.41E-08 | 19 | T5_T0 | MF | structural constituent of ribosome |
| GO:0005524 | ATP binding | 1.83E-02 | 44 | T5_T0 | MF | ATP binding |
| GO:0008094 | ATP-dependent activity, acting on DNA | 2.94E-03 | 5 | T5_T0 | MF | DNA-directed DNA polymerase activity |
| GO:0008311 | double-stranded DNA 3'-5' exodeoxyribonuclease activity | 3.44E-02 | 2 | T5_T0 | MF | endonuclease activity |
| GO:0008408 | 3'-5' exonuclease activity | 3.35E-02 | 4 | T5_T0 | MF | endonuclease activity |
| GO:0016772 | transferase activity, transferring phosphorus-containing groups | 3.44E-02 | 14 | T5_T0 | MF | DNA-directed DNA polymerase activity |
| GO:0016779 | nucleotidyltransferase activity | 2.94E-03 | 10 | T5_T0 | MF | DNA-directed DNA polymerase activity |
| GO:0016787 | hydrolase activity | 6.13E-04 | 51 | T5_T0 | MF | endonuclease activity |
| GO:0016788 | hydrolase activity, acting on ester bonds | 2.31E-03 | 15 | T5_T0 | MF | endonuclease activity |
| GO:0016813 | hydrolase activity, acting on carbon-nitrogen (but not peptide) bonds, in linear amidines | 2.34E-02 | 3 | T5_T0 | MF | hydrolase activity, acting on carbon-nitrogen (but not peptide) |
| GO:0016818 | hydrolase activity, acting on acid anhydrides, in phosphorus-containing anhydrides | 3.70E-02 | 12 | T5_T0 | MF | ATP hydrolysis activity |
| GO:0016887 | ATP hydrolysis activity | 3.61E-03 | 23 | T5_T0 | MF | ATP hydrolysis activity |
| GO:0016891 | endoribonuclease activity, producing 5'-phosphomonoesters | 3.53E-02 | 3 | T5_T0 | MF | endonuclease activity |
| GO:0017111 | ribonucleoside triphosphate phosphatase activity | 2.66E-02 | 11 | T5_T0 | MF | ATP hydrolysis activity |
| GO:0019843 | rRNA binding | 1.62E-04 | 12 | T5_T0 | MF | RNA binding |
| GO:0034061 | DNA polymerase activity | 1.04E-04 | 6 | T5_T0 | MF | DNA-directed DNA polymerase activity |
| GO:0043021 | ribonucleoprotein complex binding | 2.34E-02 | 3 | T5_T0 | MF | ribosome binding |
| GO:0043022 | ribosome binding | 2.34E-02 | 3 | T5_T0 | MF | ribosome binding |
| GO:0140097 | catalytic activity, acting on DNA | 3.80E-06 | 14 | T5_T0 | MF | DNA-directed DNA polymerase activity |
| GO:0140658 | ATP-dependent chromatin remodeler activity | 3.53E-02 | 3 | T5_T0 | MF | DNA-directed DNA polymerase activity |
| GO:0003676 | nucleic acid binding | 5.39E-20 | 83 | T30_T0 | MF | RNA binding |
| GO:0003723 | RNA binding | 1.15E-24 | 53 | T30_T0 | MF | RNA binding |
| GO:0003724 | RNA helicase activity | 4.91E-02 | 4 | T30_T0 | MF | DNA-directed 5'-3' RNA polymerase activity |
| GO:0003735 | structural constituent of ribosome | 2.73E-32 | 43 | T30_T0 | MF | structural constituent of ribosome |
| GO:0003743 | translation initiation factor activity | 1.36E-03 | 4 | T30_T0 | MF | translation initiation factor activity |
| GO:0003746 | translation elongation factor activity | 1.36E-03 | 4 | T30_T0 | MF | translation initiation factor activity |
| GO:0003899 | DNA-directed 5'-3' RNA polymerase activity | 1.39E-02 | 4 | T30_T0 | MF | DNA-directed 5'-3' RNA polymerase activity |
| GO:0003924 | GTPase activity | 1.03E-02 | 7 | T30_T0 | MF | GTPase activity |
| GO:0004519 | endonuclease activity | 9.77E-04 | 17 | T30_T0 | MF | endonuclease activity |
| GO:0005198 | structural molecule activity | 7.32E-32 | 43 | T30_T0 | MF | structural constituent of ribosome |
| GO:0008135 | translation factor activity, RNA binding | 4.02E-06 | 8 | T30_T0 | MF | translation initiation factor activity |
| GO:0016779 | nucleotidyltransferase activity | 4.30E-02 | 10 | T30_T0 | MF | DNA-directed 5'-3' RNA polymerase activity |
| GO:0019843 | rRNA binding | 4.39E-21 | 30 | T30_T0 | MF | RNA binding |
| GO:0034062 | 5'-3' RNA polymerase activity | 1.39E-02 | 4 | T30_T0 | MF | DNA-directed 5'-3' RNA polymerase activity |
| GO:0097747 | RNA polymerase activity | 1.39E-02 | 4 | T30_T0 | MF | DNA-directed 5'-3' RNA polymerase activity |
| GO:0003676 | nucleic acid binding | 8.95E-07 | 49 | T60_T0 | MF | RNA binding |
| GO:0003723 | RNA binding | 9.42E-13 | 35 | T60_T0 | MF | RNA binding |
| GO:0003735 | structural constituent of ribosome | 6.11E-15 | 27 | T60_T0 | MF | structural constituent of ribosome |
| GO:0005198 | structural molecule activity | 8.06E-15 | 27 | T60_T0 | MF | structural constituent of ribosome |
| GO:0008135 | translation factor activity, RNA binding | 6.66E-03 | 5 | T60_T0 | MF | translation initiation factor activity |
| GO:0019843 | rRNA binding | 2.72E-12 | 21 | T60_T0 | MF | RNA binding |
| GO:0003676 | nucleic acid binding | 2.93E-12 | 89 | T120_T0 | MF | RNA binding |
| GO:0003723 | RNA binding | 2.19E-10 | 44 | T120_T0 | MF | RNA binding |
| GO:0003735 | structural constituent of ribosome | 1.05E-16 | 36 | T120_T0 | MF | structural constituent of ribosome |
| GO:0003743 | translation initiation factor activity | 9.29E-03 | 4 | T120_T0 | MF | translation initiation factor activity |
| GO:0004519 | endonuclease activity | 3.68E-05 | 24 | T120_T0 | MF | endonuclease activity |
| GO:0005198 | structural molecule activity | 1.83E-16 | 36 | T120_T0 | MF | structural constituent of ribosome |
| GO:0008135 | translation factor activity, RNA binding | 1.83E-02 | 6 | T120_T0 | MF | translation initiation factor activity |
| GO:0019843 | rRNA binding | 1.29E-13 | 28 | T120_T0 | MF | RNA binding |

**Table S9.** Enriched GO terms from the Molecular Function ontology for each time point as compared to the baseline. All enriched terms were up-regulated as compared to baseline. P-values were adjusted using the Benjamini-Hochberg method. The Summary Term is the grouping used in Figure 6.

| GO ID | Description | Adjusted p-value | Gene  Count | Time | Ontology | Summary term |
| --- | --- | --- | --- | --- | --- | --- |
| GO:0005829 | cytosol | 2.23E-03 | 34 | T5_T0 | CC | cytosol |
| GO:0005840 | ribosome | 7.91E-09 | 21 | T5_T0 | CC | ribosome |
| GO:0009360 | DNA polymerase III complex | 2.34E-02 | 3 | T5_T0 | CC | excinuclease repair complex |
| GO:0009380 | excinuclease repair complex | 1.17E-02 | 3 | T5_T0 | CC | excinuclease repair complex |
| GO:0015934 | large ribosomal subunit | 4.11E-04 | 9 | T5_T0 | CC | ribonucleoprotein complex |
| GO:0015935 | small ribosomal subunit | 1.61E-02 | 6 | T5_T0 | CC | ribonucleoprotein complex |
| GO:0022625 | cytosolic large ribosomal subunit | 2.15E-04 | 9 | T5_T0 | CC | ribonucleoprotein complex |
| GO:0022626 | cytosolic ribosome | 7.99E-05 | 12 | T5_T0 | CC | cytosolic ribosome |
| GO:0032991 | protein-containing complex | 1.24E-07 | 31 | T5_T0 | CC | ribonucleoprotein complex |
| GO:0043226 | organelle | 1.56E-05 | 21 | T5_T0 | CC | ribosome |
| GO:0043228 | non-membrane-bounded organelle | 4.41E-08 | 21 | T5_T0 | CC | ribosome |
| GO:0043229 | intracellular organelle | 1.13E-05 | 21 | T5_T0 | CC | ribosome |
| GO:0043232 | intracellular non-membrane-bounded organelle | 4.41E-08 | 21 | T5_T0 | CC | ribosome |
| GO:0044391 | ribosomal subunit | 3.70E-06 | 15 | T5_T0 | CC | ribonucleoprotein complex |
| GO:1902494 | catalytic complex | 2.23E-02 | 10 | T5_T0 | CC | excinuclease repair complex |
| GO:1905347 | endodeoxyribonuclease complex | 1.17E-02 | 3 | T5_T0 | CC | excinuclease repair complex |
| GO:1905348 | endonuclease complex | 1.17E-02 | 3 | T5_T0 | CC | excinuclease repair complex |
| GO:1990391 | DNA repair complex | 1.17E-02 | 3 | T5_T0 | CC | excinuclease repair complex |
| GO:1990904 | ribonucleoprotein complex | 7.91E-09 | 21 | T5_T0 | CC | ribonucleoprotein complex |
| GO:0005618 | cell wall | 2.99E-03 | 44 | T30_T0 | CC | cell wall |
| GO:0005829 | cytosol | 9.92E-09 | 58 | T30_T0 | CC | cytosol |
| GO:0005840 | ribosome | 1.21E-33 | 46 | T30_T0 | CC | ribonucleoprotein complex |
| GO:0015934 | large ribosomal subunit | 2.31E-13 | 19 | T30_T0 | CC | ribonucleoprotein complex |
| GO:0015935 | small ribosomal subunit | 1.21E-10 | 15 | T30_T0 | CC | ribonucleoprotein complex |
| GO:0022625 | cytosolic large ribosomal subunit | 6.02E-13 | 18 | T30_T0 | CC | ribonucleoprotein complex |
| GO:0022626 | cytosolic ribosome | 9.79E-20 | 28 | T30_T0 | CC | cytosolic ribosome |
| GO:0022627 | cytosolic small ribosomal subunit | 1.08E-05 | 9 | T30_T0 | CC | ribonucleoprotein complex |
| GO:0030312 | external encapsulating structure | 3.17E-03 | 44 | T30_T0 | CC | cell wall |
| GO:0032991 | protein-containing complex | 6.34E-16 | 50 | T30_T0 | CC | ribonucleoprotein complex |
| GO:0043226 | organelle | 1.58E-23 | 48 | T30_T0 | CC | intracellular non-membrane-bounded organelle |
| GO:0043228 | non-membrane-bounded organelle | 3.59E-30 | 46 | T30_T0 | CC | intracellular non-membrane-bounded organelle |
| GO:0043229 | intracellular organelle | 5.39E-24 | 48 | T30_T0 | CC | intracellular non-membrane-bounded organelle |
| GO:0043232 | intracellular non-membrane-bounded organelle | 3.59E-30 | 46 | T30_T0 | CC | intracellular non-membrane-bounded organelle |
| GO:0044391 | ribosomal subunit | 3.01E-24 | 34 | T30_T0 | CC | ribonucleoprotein complex |
| GO:1990904 | ribonucleoprotein complex | 2.07E-33 | 46 | T30_T0 | CC | ribonucleoprotein complex |
| GO:0005829 | cytosol | 1.05E-03 | 38 | T60_T0 | CC | cytosol |
| GO:0005840 | ribosome | 1.52E-15 | 29 | T60_T0 | CC | ribonucleoprotein complex |
| GO:0015934 | large ribosomal subunit | 2.12E-06 | 12 | T60_T0 | CC | ribonucleoprotein complex |
| GO:0015935 | small ribosomal subunit | 1.18E-05 | 10 | T60_T0 | CC | ribonucleoprotein complex |
| GO:0022625 | cytosolic large ribosomal subunit | 8.31E-07 | 12 | T60_T0 | CC | ribonucleoprotein complex |
| GO:0022626 | cytosolic ribosome | 7.17E-11 | 19 | T60_T0 | CC | cytosolic ribosome |
| GO:0022627 | cytosolic small ribosomal subunit | 4.68E-03 | 6 | T60_T0 | CC | ribonucleoprotein complex |
| GO:0032991 | protein-containing complex | 8.95E-07 | 31 | T60_T0 | CC | ribonucleoprotein complex |
| GO:0043226 | organelle | 1.91E-12 | 32 | T60_T0 | CC | intracellular non-membrane-bounded organelle |
| GO:0043228 | non-membrane-bounded organelle | 2.77E-14 | 29 | T60_T0 | CC | intracellular non-membrane-bounded organelle |
| GO:0043229 | intracellular organelle | 1.10E-12 | 32 | T60_T0 | CC | intracellular non-membrane-bounded organelle |
| GO:0043232 | intracellular non-membrane-bounded organelle | 2.77E-14 | 29 | T60_T0 | CC | intracellular non-membrane-bounded organelle |
| GO:0044391 | ribosomal subunit | 3.26E-12 | 22 | T60_T0 | CC | ribonucleoprotein complex |
| GO:1990904 | ribonucleoprotein complex | 1.52E-15 | 29 | T60_T0 | CC | ribonucleoprotein complex |
| GO:0005840 | ribosome | 1.05E-16 | 38 | T120_T0 | CC | ribonucleoprotein complex |
| GO:0015934 | large ribosomal subunit | 6.44E-08 | 17 | T120_T0 | CC | ribonucleoprotein complex |
| GO:0015935 | small ribosomal subunit | 5.59E-07 | 14 | T120_T0 | CC | ribonucleoprotein complex |
| GO:0022625 | cytosolic large ribosomal subunit | 1.23E-07 | 16 | T120_T0 | CC | ribonucleoprotein complex |
| GO:0022626 | cytosolic ribosome | 1.34E-12 | 26 | T120_T0 | CC | cytosolic ribosome |
| GO:0022627 | cytosolic small ribosomal subunit | 3.55E-04 | 9 | T120_T0 | CC | ribonucleoprotein complex |
| GO:0032991 | protein-containing complex | 1.04E-06 | 45 | T120_T0 | CC | ribonucleoprotein complex |
| GO:0043226 | organelle | 3.02E-10 | 40 | T120_T0 | CC | intracellular non-membrane-bounded organelle |
| GO:0043228 | non-membrane-bounded organelle | 1.28E-14 | 38 | T120_T0 | CC | intracellular non-membrane-bounded organelle |
| GO:0043229 | intracellular organelle | 1.60E-10 | 40 | T120_T0 | CC | intracellular non-membrane-bounded organelle |
| GO:0043232 | intracellular non-membrane-bounded organelle | 1.28E-14 | 38 | T120_T0 | CC | intracellular non-membrane-bounded organelle |
| GO:0044391 | ribosomal subunit | 8.05E-15 | 31 | T120_T0 | CC | ribonucleoprotein complex |
| GO:1990904 | ribonucleoprotein complex | 1.05E-16 | 38 | T120_T0 | CC | ribonucleoprotein complex |

**Table S10.** Enriched GO terms for sequential comparisons at 30 minutes versus 5 minutes and 120 minutes versus 60 minutes. There were no significantly enriched terms at 60 minutes versus 30 minutes. P-values were adjusted using the Benjamini-Hochberg method.

| ID | Description | Adjusted p-value | Gene  Count | Comparison |
| --- | --- | --- | --- | --- |
| Up-regulated |  |  |  |  |
| GO:0006633 | fatty acid biosynthetic process | 3.83E-02 | 4 | T30_T5 |
| GO:0004519 | endonuclease activity | 2.54E-09 | 13 | T120_T60 |
| GO:0003676 | nucleic acid binding | 9.55E-05 | 18 | T120_T60 |
| Down-regulated |  |  |  |  |
| GO:0006259 | DNA metabolic process | 9.45E-08 | 13 | T30_T5 |
| GO:0006260 | DNA replication | 1.00E-06 | 9 | T30_T5 |
| GO:0004386 | helicase activity | 4.01E-06 | 8 | T30_T5 |
| GO:0006974 | cellular response to DNA damage stimulus | 1.44E-05 | 9 | T30_T5 |
| GO:0003887 | DNA-directed DNA polymerase activity | 1.02E-04 | 5 | T30_T5 |
| GO:0004527 | exonuclease activity | 1.21E-04 | 6 | T30_T5 |
| GO:0006281 | DNA repair | 1.68E-04 | 8 | T30_T5 |
| GO:0140097 | catalytic activity, acting on DNA | 2.21E-04 | 7 | T30_T5 |
| GO:0090305 | nucleic acid phosphodiester bond hydrolysis | 2.72E-04 | 7 | T30_T5 |
| GO:0004518 | nuclease activity | 2.86E-04 | 7 | T30_T5 |
| GO:0008094 | ATP-dependent activity, acting on DNA | 4.96E-04 | 4 | T30_T5 |
| GO:0006950 | response to stress | 1.26E-03 | 10 | T30_T5 |
| GO:0033554 | cellular response to stress | 1.76E-03 | 8 | T30_T5 |
| GO:0090304 | nucleic acid metabolic process | 4.51E-03 | 13 | T30_T5 |
| GO:0006310 | DNA recombination | 5.77E-03 | 5 | T30_T5 |
| GO:0004748 | ribonucleoside-diphosphate reductase activity, thioredoxin disulfide as acceptor | 5.77E-03 | 2 | T30_T5 |
| GO:0005971 | ribonucleoside-diphosphate reductase complex | 5.77E-03 | 2 | T30_T5 |
| GO:0016728 | oxidoreductase activity, acting on CH or CH2 groups, disulfide as acceptor | 5.77E-03 | 2 | T30_T5 |
| GO:0061731 | ribonucleoside-diphosphate reductase activity | 5.77E-03 | 2 | T30_T5 |
| GO:0034061 | DNA polymerase activity | 6.90E-03 | 3 | T30_T5 |
| GO:0016788 | hydrolase activity, acting on ester bonds | 7.18E-03 | 7 | T30_T5 |
| GO:0016818 | hydrolase activity, acting on acid anhydrides, in phosphorus-containing anhydrides | 7.18E-03 | 7 | T30_T5 |
| GO:0071897 | DNA biosynthetic process | 8.46E-03 | 3 | T30_T5 |
| GO:0008408 | 3'-5' exonuclease activity | 1.06E-02 | 3 | T30_T5 |
| GO:0051716 | cellular response to stimulus | 1.06E-02 | 8 | T30_T5 |
| GO:0004519 | endonuclease activity | 1.17E-02 | 6 | T30_T5 |
| GO:0017111 | ribonucleoside triphosphate phosphatase activity | 1.22E-02 | 6 | T30_T5 |
| GO:0006261 | DNA-templated DNA replication | 1.84E-02 | 3 | T30_T5 |
| GO:0050896 | response to stimulus | 1.84E-02 | 11 | T30_T5 |
| GO:0016787 | hydrolase activity | 1.84E-02 | 17 | T30_T5 |
| GO:0009380 | excinuclease repair complex | 1.84E-02 | 2 | T30_T5 |
| GO:0016725 | oxidoreductase activity, acting on CH or CH2 groups | 1.84E-02 | 2 | T30_T5 |
| GO:1905347 | endodeoxyribonuclease complex | 1.84E-02 | 2 | T30_T5 |
| GO:1905348 | endonuclease complex | 1.84E-02 | 2 | T30_T5 |
| GO:1990391 | DNA repair complex | 1.84E-02 | 2 | T30_T5 |
| GO:0016462 | pyrophosphatase activity | 2.15E-02 | 6 | T30_T5 |
| GO:1902494 | catalytic complex | 2.32E-02 | 5 | T30_T5 |
| GO:0016817 | hydrolase activity, acting on acid anhydrides | 2.32E-02 | 6 | T30_T5 |
| GO:0032553 | ribonucleotide binding | 3.25E-02 | 11 | T30_T5 |
| GO:0006139 | nucleobase-containing compound metabolic process | 3.27E-02 | 14 | T30_T5 |
| GO:0009263 | deoxyribonucleotide biosynthetic process | 3.57E-02 | 2 | T30_T5 |
| GO:0140658 | ATP-dependent chromatin remodeler activity | 3.57E-02 | 2 | T30_T5 |
| GO:0097367 | carbohydrate derivative binding | 3.57E-02 | 11 | T30_T5 |
| GO:0044260 | cellular macromolecule metabolic process | 4.53E-02 | 14 | T30_T5 |
| GO:0003676 | nucleic acid binding | 4.85E-02 | 12 | T30_T5 |
